# Contrasting population genetic structure among freshwater-resident and anadromous lampreys: the role of demographic history, differential dispersal and anthropogenic barriers to movement

**DOI:** 10.1111/mec.13112

**Published:** 2015-03-06

**Authors:** Fiona S A Bracken, A Rus Hoelzel, John B Hume, Martyn C Lucas

**Affiliations:** * School of Biological and Biomedical Sciences, Durham UniversityDurham, DH1 3LE, UK; † Institute of Biodiversity, Animal Health & Comparative Medicine, University of GlasgowGlasgow, UK

**Keywords:** anadromy, barriers to migration, *Lampetra*, life history, microsatellite, speciation

## Abstract

The tendency of many species to abandon migration remains a poorly understood aspect of evolutionary biology that may play an important role in promoting species radiation by both allopatric and sympatric mechanisms. Anadromy inherently offers an opportunity for the colonization of freshwater environments, and the shift from an anadromous to a wholly freshwater life history has occurred in many families of fishes. Freshwater-resident forms have arisen repeatedly among lampreys (within the Petromyzontidae and Mordaciidae), and there has been much debate as to whether anadromous lampreys, and their derived freshwater-resident analogues, constitute distinct species or are divergent ecotypes of polymorphic species. Samples of 543 European river lamprey *Lampetra fluviatilis* (mostly from anadromous populations) and freshwater European brook lamprey *Lampetra planeri* from across 18 sites, primarily in the British Isles, were investigated for 13 polymorphic microsatellite DNA loci, and 108 samples from six of these sites were sequenced for 829 bp of mitochondrial DNA (mtDNA). We found contrasting patterns of population structure for mtDNA and microsatellite DNA markers, such that low diversity and little structure were seen for all populations for mtDNA (consistent with a recent founder expansion event), while fine-scale structuring was evident for nuclear markers. Strong differentiation for microsatellite DNA loci was seen among freshwater-resident *L. planeri* populations and between *L. fluviatilis* and *L. planeri* in most cases, but little structure was evident among anadromous *L. fluviatilis* populations. We conclude that postglacial colonization founded multiple freshwater-resident populations with strong habitat fidelity and limited dispersal tendencies that became highly differentiated, a pattern that was likely intensified by anthropogenic barriers.

## Introduction

Although the abandonment of migration remains a poorly understood aspect of evolutionary biology, there is evidence to suggest that this phenomenon might act as an initiator for adaptive radiation (Bell & Andrews 1997; Winker 2000; Räsänen & Hendry 2008; Langerhans & Riesch 2013). Differences in life history traits between resident and migrant individuals can be thought of as adaptive behaviours that act to increase growth, survival rate, fecundity and egg quality. This is reflected in the fitness outcomes of both life history strategies, with residency favoured when the cost of migration exceeds the benefits of doing so, particularly in terms of growth potential and mortality risk before reproduction (Fryxell & Sinclair 1988; Bell & Andrews 1997; Dingle 2006; Brönmark *et al*. 2008; Shaw & Couzin 2013).

Anadromy, which involves reproduction in freshwater and the majority of growth in the marine environment, is a distinctive migratory trait that is recognized in 18 fish families and 120 species (McDowall 1997; Chapman *et al*. 2012). Anadromy inherently offers an opportunity to colonize previously unexploited freshwater environments, and the shift from an anadromous to a wholly freshwater life history has occurred repeatedly in many taxa of fishes (e.g. Petromyzontiformes, Salmonidae, Gasterosteidae; Potter 1980; Taylor *et al*. 1996; Lucas & Baras 2001). Glacial cycles may have supported the evolution of wholly freshwater forms by either blocking migration routes and preventing anadromy or, upon deglaciation, making available new habitat and food resources that are inaccessible through freshwater but easily reached by anadromous fish (Bell & Andrews 1997; Lee & Bell 1999).

The extent to which anadromy is obligatory varies among species. Many populations of anadromous fishes contain a component that does not migrate to sea and instead remains in freshwater where they mature and spawn. In some cases, they may subsequently move little, but in other cases migrate between distinct freshwater habitats (potamodromy)**,** often reproducing with their anadromous conspecifics (Lucas & Baras 2001; McDowall 2001). ‘Partial migration’ is the term coined for this resident-migratory dimorphism within populations (Chapman *et al*. 2011), and it is widespread in mammals, invertebrates, birds (Lundberg 1988; Jahn *et al*. 2010) and fishes (Olsson & Greenberg 2004; Brodersen *et al*. 2008; Kerr *et al*. 2009; Chapman *et al*. 2012).

Incipient speciation in these systems may be promoted through both allopatric and sympatric mechanisms (Chapman *et al*. 2011). Reduced gene flow between migrants and freshwater-residents breeding in allopatry could promote differentiation by genetic drift or local adaptation. Conversely, population differentiation is limited by the large-scale dispersal capacity of migrants, resulting in a greater chance of panmixia (Hoarau *et al*. 2002; Coltman *et al*. 2007). Migratory populations that exhibit philopatry, or habitat fidelity, however, can maintain discrete genetic differences between populations within species. For example, anadromous Atlantic salmon (*Salmo salar*) undergo extended oceanic migrations, yet exhibit significant local adaptation and substantial reproductive isolation between populations owing to precise philopatry and a high homing fidelity to their natal river or tributary (Taylor 1991).

In contrast to anadromous salmonids, anadromous lampreys (Petromyzontiformes) generally show very low interpopulation differentiation across geographically distant river systems (Almada *et al*. 2008; Goodman *et al*. 2008) and have been shown to use pheromones released by stream dwelling larvae as partial cues to find suitable spawning habitats (Fine *et al*. 2004). An evolutionary trend among lampreys is the occurrence in most genera of ‘paired species’ (Zanandrea 1959), whereby larvae are morphologically indistinguishable, while the adults of two putative species adopt either a nonparasitic freshwater-resident or a parasitic life history which can be either potamodromous or anadromous.

Nonparasitism has arisen repeatedly among lampreys (Docker 2009) and even within species (Espanhol *et al*. 2007), suggesting that feeding type is plastic and nonparasitic lineages may be polyphyletic (Docker 2009; Renaud *et al*. 2009). Nonetheless, there has been much controversy about the taxonomic status of many paired lamprey species (Zanandrea 1959; Hardisty 1986a; Schreiber & Engelhorn 1998; Youson & Sower 2001; Gill *et al*. 2003; Renaud *et al*. 2009; Docker *et al*. 2012). Although various studies have found little genetic differentiation between lamprey paired species (Docker *et al*. 1999; Yamazaki *et al*. 2006; Espanhol *et al*. 2007; Blank *et al*. 2008; Lang *et al*. 2009), Mateus *et al*. (2013b) found significant differentiation between sympatric European river and brook lamprey populations in Portugal based on nuclear genomic data, and Taylor *et al*. (2012) report differentiation between anadromous and freshwater-resident parasitic lampreys in British Columbia based on eight microsatellite DNA loci.

Here, we explore the population genetics of the anadromous European river lamprey (*Lampetra fluviatilis* L. 1758) and its nonparasitic freshwater-resident derivative the European brook lamprey (*Lampetra planeri* Bloch 1784), together with several *L. fluviatilis* populations that comprise potamodromous individuals that migrate within freshwater only (i.e. freshwater-residents; Maitland *et al*. 1994; Inger *et al*. 2010). We use a combination of mtDNA and microsatellite nuclear DNA markers to test the hypothesis that the postglacial expansion of anadromous *L. fluviatilis* during the Holocene prompted the establishment of multiple freshwater-resident *L.planeri* populations that subsequently became genetically differentiated. We also investigated the possibility that anthropogenic barriers are isolating lamprey populations and provide a robust quantitative assessment of this. In some freshwater fishes, the fragmentation of habitats by dams can promote genetic differentiation between the upstream and downstream populations resulting from the reduction of gene flow, often compounded by founder effects and subsequent genetic drift (Yamamoto *et al*. 2004; Palkovacs *et al*. 2008). Population divergence and dispersal at local to catchment scales were examined enabling inference about population connectivity and evolutionary viability, which may indicate important applications in conservation management (Latta 2008) and enhance our understanding of the systematics of these ancient fish.

## Methods

### Sampling and DNA isolation

**Fig 1:**
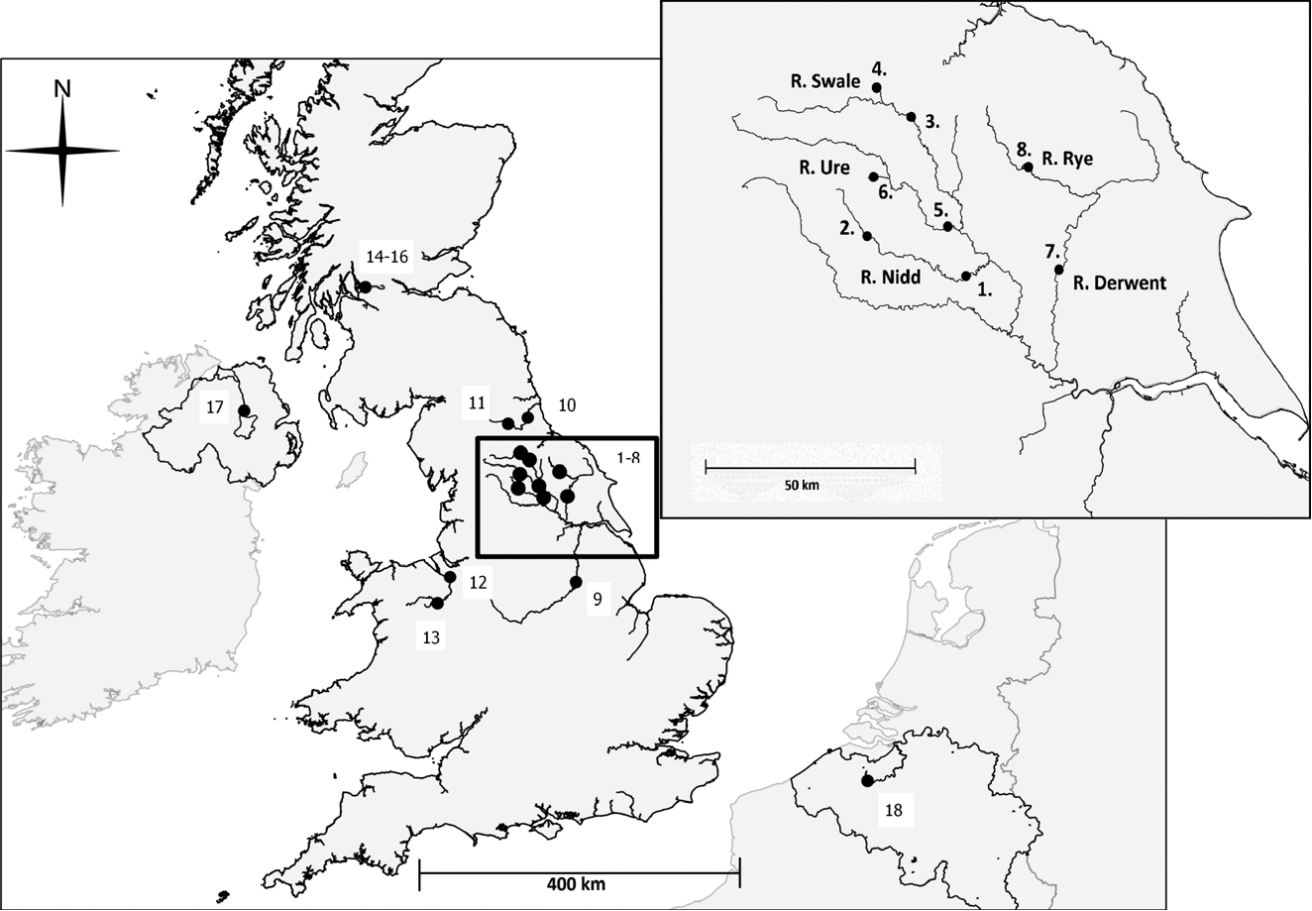
Map showing location of sampling sites 1–18 (see Table S1, Supporting information for detail). Inset is a detailed map of part of the Ouse subcatchment of the Humber catchment, showing sampling locations. Only sampled rivers are shown.

Tissue samples were collected across a total of 18 sites (Fig.1, Table S1, Supporting information). Unlike, for example, in some Baltic regions (Sjöberg 2011), there is no evidence or likelihood of historical stocking or translocation of lampreys at any of these sites. MtDNA loci were examined in *n* = 108 individuals from six sites including two paired sites (i.e. where *Lampetra planeri* and *Lampetra fluviatilis* were obtained from the same river; Table S1, Supporting information, Table 1, Fig.1). For microsatellite loci, 543 samples were collected from 18 sites, including seven paired sites (PS; Table S1, Supporting information, Fig.1). One of these paired sites also included a freshwater-resident *L. fluviatilis* population (PS7; Loch Lomond, Scotland, Table S1, Supporting information). Three additional sites for *L. fluviatilis* were also included in the analysis (sites 9, 17, 18, Table S1, Supporting information, Fig.1); one of which is a freshwater-resident population of *L. fluviatilis* in the River Bann (site 17). In Loch Lomond (PS7 in Table S1, Supporting information; Scotland), all three ‘ecotypes’ (i.e. *L. planeri*, *L. fluviatilis* and freshwater-resident *L. fluviatilis*) are truly sympatric; however, in all other paired sites, *L. planeri* samples were obtained upstream (within the same river) of anadromous *L. fluviatilis* populations, which were usually separated by migration barriers (Table S2, Supporting information). It should also be noted that the location from which the River Swale *L. planeri* samples were obtained is a spawning site for both *L. planeri* and sometimes *L. fluviatilis*.

**Table 1:** Diversity indices for MtDNA ATPase

Population	Site no.	Country	*N*	*H*	π	*h*	*D*	*D*_*P*_	*F*s	*F*s_*P*_
Bann (Lf Res)	17	N. Ireland	20	3	0.0002 ± 0.0003	0.1947	−1.5128	0.059	−1.1801	0.015
Scheldt (Lf)	18	Belgium	20	10	0.0012 ± 0.0009	0.7105	−2.0976	0.006	−8.7029	0
Nidd (Lf)	1	England	17	2	0.0001 ± 0.0002	0.1176	−1.1639	0.125	−0.7484	0.092
Nidd (Lp)	2	England	18	1	0	0	0	N.A.	0	N.A.
Dee (Lf)	12	Wales	16	4	0.0006 ± 0.0006	0.3500	−1.8309	0.015	−1.7904	0.014
Dee (Lp)	13	Wales	17	2	0.0003 ± 0.0004	0.2206	−0.4913	0.264	0.0353	0.255
All			108	16	0.0007 ± 0.0006	0.4907	−2.1898	0	−18.4452	0

MtDNA analysis was performed on only a subset of the 543 lampreys and 18 sites used for the microsatellite analysis. The ‘Site No.’ column corresponds to the site numbers in Fig.1 and Table S1 (Supporting information).

*N =* Sample size, *H =* number of haplotypes*,* π = nucleotide diversity, *h* = haplotype diversity, *D* = Tajima's *D*, *D*_*P*_ = Tajima's *D P*-value, *F*s = Fu's *F*, *F*s_*P*_ = Fu's *F P*-value, and Lf = *L. fluviatilis,* Lp = *L. planeri,* and Lf Res = freshwater-resident population of *L. fluviatilis*.

Samples were obtained by hand-netting, electro-fishing and the utilization of static double-funnel traps to capture spawning, and upstream-migrating lampreys (Table S1, Supporting information). Both *L. fluviatilis* and *L. planeri* were sampled where they were found to be locally abundant prior to the spawning period and so were, in most cases, captured in the vicinity of their spawning grounds. *L. planeri* were normally captured in the upstream reaches of rivers where they were abundant, and in all cases, except at the Endrick Water, Loch Lomond were sampled upstream of the *L. fluviatilis* spawning areas. Only adult and juvenile lampreys, unambiguously identifiable to species, were included in this study. Adult anadromous, and freshwater-resident *L. fluviatilis* (e.g. Loch Lomond, Morris 1989; and the R. Bann, Goodwin *et al*. 2006), as well as nonparasitic *L. planeri* can be separated using standard lamprey taxonomic characteristics (Renaud 2011). Individuals were identified and measured under anaesthesia (MS-222, 0.1 g/L) using a field key (Gardiner 2003), and fin clips taken from the second dorsal fin were stored in 20% DMSO saturated NaCl solution (Amos & Hoelzel 1991). Total genomic DNA was extracted from samples using a proteinase K digestion procedure followed by the standard phenol–chloroform method and stored at −20 °C.

### Amplification and sequencing of mitochondrial DNA

The PCR primers ATPfor and ATPrev (Espanhol *et al*. 2007) were used to amplify 838 bp of the mitochondrial gene ATPase subunits 6 and 8. This locus was chosen to facilitate comparison with previous data from Espanhol *et al*. (2007) and Mateus *et al*. (2011). Each 20 μL reaction contained 1.2 μL (final conc. 1.5 mm) MgCl_2_, 2 μL dNTPs (2.0 mm), 0.2 μL of each primer (10 mm), 4 μL of Colorless GoTaq® Reaction Buffer (Promega), 0.1 μL GoTaq DNA polymerase (Promega) and 1 μL of template DNA. Cycle conditions were as follows: initial denaturation at 94 °C for 3 min, followed by 30 cycles of; denaturation at 94 °C for 1 min, annealing temperature 57.1 °C for 1 min and extension at 72 °C for 2 min; and followed by a final extension at 72 °C for 2 min. The resulting PCR products were purified using the Qiagen PCR Purification kit and sequenced using an ABI PRISM 3730 DNA Analyser (DBS genomics Durham University).

### Amplification and genotyping of microsatellites

Thirteen recently developed polymorphic microsatellite loci were used to examine genetic differentiation among and between all *L. fluviatilis* and *L. planeri* populations. Eight microsatellite primers developed for European *Lampetra* (Lp-003, Lp-006, Lp-009, Lp-018, Lp-027, Lp-028, Lp-046 and Lp-045; Gaigher *et al*. 2013), one primer set developed for *Lampetra richardsoni* (Lri-5; Luzier *et al*. 2010), and four microsatellite primers developed in this study (using the protocol described in White *et al*. 2010) and optimized for European *Lampetra* species (Lamper_1, Lamper_2, Lamper_3, Lamper_4) were included (Table S3, Supporting information).

Microsatellite loci were multiplex amplified using a Qiagen Multiplex kit. Thermal cycler conditions were as follows: initial denaturation at 95 °C for 15 min; followed by 35 cycles of denaturation at 94 °C for 30 s, annealing temperature 60 °C for 90 s and extension at 72 °C for 60 s; and followed by a final extension at 60 °C for 30 min. PCR products were genotyped on a 3730 ABI DNA Analyser (DBS Genomics, Durham, UK) and visualized with Geneious VR6 (Biomatters). Microsatellite loci were tested for null alleles, large allele dropout and scoring errors due to stutter peaks using microchecker 2.2.3 (van Oosterhout *et al*. 2004). The program arlequin 3.5 (Excoffier & Lischer 2010) was then used to test deviation from Hardy–Weinberg equilibrium. Tests for linkage disequilibrium were carried out for each pair of loci using an exact test based on a Markov chain method as implemented in genepop 4.2 (Raymond & Rousset 1995; Rousset 2008). The program Lositan (Antao *et al*. 2008) was used to test for outliers indicating positive or balancing selection (using a forced neutral mean *F*_ST_, a confidence interval of 0.99 and false discovery of 0.1), and no loci with evidence for selection were found.

### Genetic diversity and structure

MtDNA sequences were aligned manually using geneious vR6 (Biomatters). The program dnasp 10.4.9 (Rozas *et al*. 2003) was then used to calculate mitochondrial DNA polymorphism estimated as haplotypic diversity (Nei & Tajima 1981) and nucleotide diversity (Nei 1987). To determine the level of genetic differentiation between pairs of populations, *F*-statistics (Weir & Cockerham 1984) were calculated for mtDNA and microsatellite DNA loci using arlequin version 3.5. Significance was tested using 1000 permutations. arlequin was also used to calculate Fu's *F*, Tajima's *D* and mismatch distributions. We estimated the putative time of population expansion from the mismatch distribution using the statistic tau (τ; Rogers & Harpending 1992). Substitution rate was estimated after Ho *et al*. (2007) who suggest an average of ∼50% per site per million years for the control region, based on recent evolutionary time frames, although of course this varies among species. The substitution rate for the control region can be ten times faster than the rest of the mitochondrial genome (McMillan & Palumbi 1997). Therefore, 5% per site per million years was used as a rough estimate for ATPase. Mutation rates of 1% and 10% per million years were also used to illustrate the effect that the rate of divergence will have in the expansion times. The relationship between haplotypes was investigated using a median-joining network (MJN) constructed using the program network 3.1.1.1 (Bandelt *et al*. 1999) and epsilon values of 0, 10, 20 and 30 were tested.

For microsatellite DNA data, allelic richness for each locus and population and *F*_IS_ (inbreeding coefficient) were calculated using the program fstat 2.9.3 (Goudet 1995). structure 2.0 was used to assign individuals by genotype to a putative number of populations (*K*; Pritchard *et al*. 2000). *ΔK*, a measure of the second order rate of change in the likelihood of *K* (Evanno *et al*. 2005), was calculated using structure Harvester (Earl & vonHoldt 2012) to assess the highest hierarchical level of structure. Four independent runs for each *K* value were performed at 2 000 000 Markov chain Monte Carlo (MCMC) repetitions and 500 000 burn-in using no population prior information and assuming correlated allele frequencies and admixture. structure was also used with a location prior (LOCPRIOR) to clarify population structure within the Loch Lomond system (Hubisz *et al*. 2009). Burn-in and run lengths were the same as for runs without prior population information. Due to the large number of putative population subdivisions, subsamples were compared by region to increase resolution, in addition to an analysis involving all regions. Full-sibling pairs within a sampling site (for the five localities where there are populations of both putative species: Wear, Dee, Derwent, Nidd & Ure) were identified using the maximum-likelihood method in colony version 2.0.1.1 with male and female polygamy permitted and a medium run length (Jones & Wang 2010).

Patterns of microsatellite differentiation were subsequently examined using a factorial correspondence analysis (FCA) implemented in genetix 4.05.2 (Belkhir *et al*. –2004^1996^), which gives a visual representation of individual genotype clustering. A test for a positive association between genetic [*F*_ST_/(1 − *F*_ST_)] and geographic distances [Isolation by distance (IBD)] based on microsatellite DNA loci was carried out using a Mantel test (10 000 permutations) in genepop v4.2. Geographic distances were calculated between sample sites using linear referencing tools in Quantum GIS (Lisboa). A Mantel test was also carried out to test for association between genetic distances and number of physical barriers (defined as any anthropogenic feature larger than 0.5 m height at base river level which reaches the full width of the river) between sample sites. The 0.5 m value was subjective, based on the fact that many structures of this height or greater generate discrete water level differences (upstream–downstream) at base flows, on published and unpublished data on the impact of different height potential barriers on lamprey movement (*L. fluviatilis*, Lucas *et al*. 2009; *L. planeri*, M. C. Lucas personal observation), and on our ability to identify potential barriers in field surveys and databases. Only river systems for which information on barriers was available were utilized in the Mantel tests (including the Dee, Wear and all rivers within the Ouse subcatchment, excluding the Swale due to the low sample size attained for *L. planeri*).

migrate-n (v 3.2.6) was used to estimate levels of historical gene flow between populations (Beerli & Felsenstein 2001; Beerli 2006; Beerli & Palczewski 2010). Pairwise comparisons were carried out between putative species (i.e. *L. fluviatilis* and *L. planeri*) at six locations (Wear, Dee, Lomond, Nidd, Ure, Derwent), of which the latter three are all tributaries in the same river catchment, where samples from both species were available. To implement Bayesian inference in MIGRATE-N, the Brownian motion approximation was selected with an MCMC search of 100 000 burn-in steps followed by 5 000 000 steps with parameters recorded every 100 steps; exponential prior on theta (min: 0, mean: 30, max: 60); and an exponential prior on migration (min: 0, mean: 650 max: 1300). migrate-n was run with parameter values starting from *F*_ST_-based estimates, and the distribution of parameter values was compared across runs to ensure overlap of 95% CI. bayesass 1.3 (Wilson & Rannala 2003) was used to estimate the magnitude and directionality of contemporary gene flow between *L. fluviatilis* and *L. planeri*. Pairwise comparisons were carried out for the same six locations that were used in the migrate-n analysis. In contrast to migrate-n, bayesass estimates all pairwise migration rates rather than a user-defined migration matrix and provides unidirectional estimates of migration for each population pair. bayesass does not assume a migration–drift equilibrium, an assumption that is frequently violated in natural populations (Whitlock & McCauley 1999). A total of 10 000 000 MCMC iterations were run of which 1 000 000 were for the burn-in. All other options were left at their default settings. Five to 10 runs with a different starting point were performed for each population pair and results are given as means. The program tracer version 1.5 (Rambaut & Drummond 2007) was used as a method to qualitatively assess MCMC convergence.

## Results

### MtDNA

**Fig 2:**
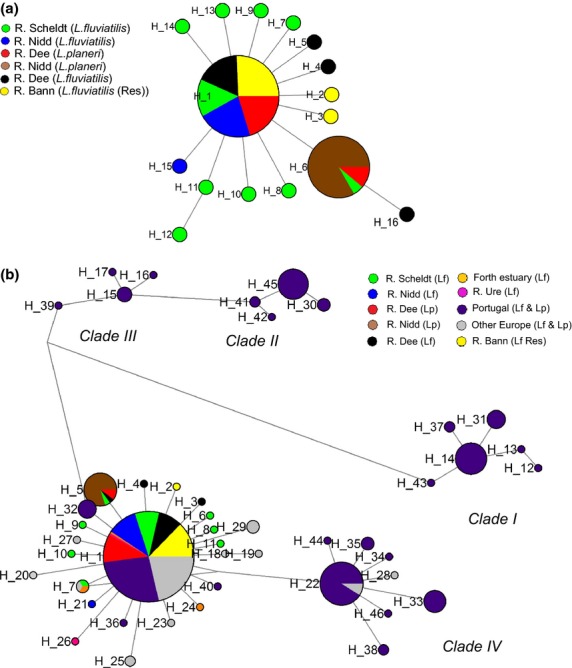
(a) Median-joining network showing 16 haplotypes found from 108 samples of *Lampetra* at six sampling locations. Note that Bann (Lf) is a freshwater-resident *L. fluviatilis* population. Lf = anadromous *L. fluviatilis*, Lp = *L. planeri* and Lf Res = freshwater-resident population of *L. fluviatilis*. Details of the sample locations are given in Table S1 (Supporting information). (b) Forty-six haplotypes from combined studies comprising of both *L. fluviatilis* and *L. planeri*. Circled groups show correspondence with clades identified in Mateus *et al*. (2011). Clades I–III consist of freshwater-resident *L. planeri* (but see Mateus *et al*. 2013b) with restricted distribution, and clade IV contains both freshwater-resident Lp and anadromous Lf with a wider distribution along with haplotypes identified in Espanhol *et al*. (2007) from France, Sweden and Germany (Lp and Lf H22) and France (Lp H28). Please see the open-access online paper for a colour version of this figure.

ATPase subunits 6 and 8 were sequenced and haplotypes determined for 108 lampreys (*Lampetra fluviatilis* and *Lampetra planeri*) from six sampling sites (Table 1). Over all populations, haplotype and nucleotide diversity were low, with freshwater-resident populations of both *L. planeri* and *L. fluviatilis* generally exhibiting lower haplotype and nucleotide diversity than the anadromous *L. fluviatilis* populations. Both Tajima's *D* and Fu's *F* were negative and highly significant (Table 1), consistent with a population expansion (e.g. after a bottleneck) or a selective sweep. Using the value of *tau,* which was 0.673 (Fig. S1, Supporting information), an expansion time of 16 263 (10 182–26 952; 95% CI) years ago was calculated using the mutation rate of 5% per million years. Using mutation rates of 1% and 10%, expansion times would be 81 182 and 8118 years ago, respectively. Sixteen haplotypes were observed, with private haplotypes found only in the *L. planeri* population from the River Nidd and no species-specific lineages (see median-joining network in Fig.2a). *F*_ST_ values between sites ranged from 0.01955 to 0.94093 with only *F*_ST_ values associated with the Nidd (*L. planeri*) being statistically significant (*P* < 0.0001; Table 2).

**Table 2:** Matrix of pairwise *F*_ST_ values for mtDNA analysis of six populations of *Lampetra*

	Bann (Lf Res)	Dee (Lf)	Nidd (Lp)	Scheldt (Lf)	Nidd (Lf)
Dee (Lf)	0.0033				
Nidd (Lp)	0.8534	0.7717			
Scheldt (Lf)	0.0074	−0.0001	0.5702		
Nidd (Lf)	−0.0038	0.0024	0.9409	0.0082	
Dee (Lp)	−0.0258	−0.0196	0.8676	0.0024	0.0417

Significant *F*_ST_ values [i.e. all *F*_ST_ values associated with Nidd (Lp)] are highlighted in grey (*P* < 0.0001).

Lf = *Lampetra fluviatilis*, Lp = *Lampetra planeri* and Lf Res = freshwater-resident population of *L. fluviatilis*.

A network showing the European haplotype distribution, incorporating data from Espanhol *et al*. (2007) and Mateus *et al*. (2011), revealed 46 haplotypes with Portuguese populations being visibly further removed from the majority of other samples (Fig.2b). Identified lineages were concordant with those reported by Mateus *et al*. (2011), and as observed by Espanhol *et al*. (2007), not species specific. Clades I, II and III were considered to be composed of adult *L. planeri* (Mateus *et al*. 2011; now regarded as three cryptic species, *L. alavariensis*, *L. auremensis* and *L. lusiticanica*, Mateus *et al*. 2013a) and larvae of unknown specific status, while clade IV comprises *L. planeri*, anadromous and freshwater-resident *L. fluviatilis* adults and larvae.

### Microsatellite analysis

**Fig 3:**
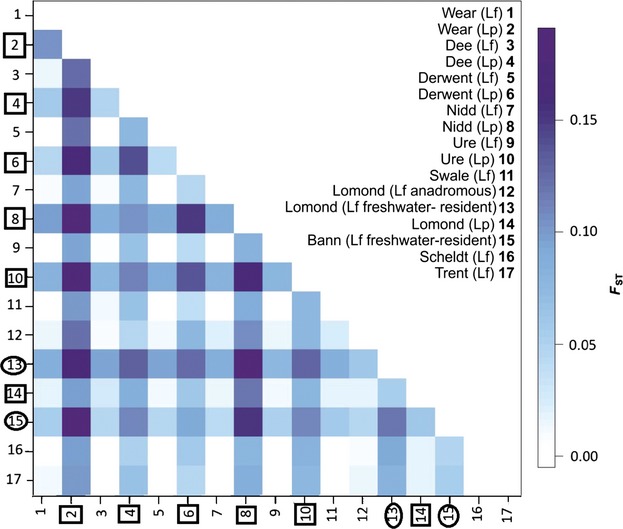
Matrix of pairwise *F*_ST_ values using 13 microsatellite loci, for all *Lampetra* populations sampled. Lf = anadromous *L. fluviatilis*, Lp = *L. planeri* and Lf Res = freshwater-resident population of *L. fluviatilis*. Table showing the actual values is included in Supporting information (Table S5). Numbers on axes are marked with a square to represent *L. planeri* and a circle to represent freshwater-resident *L. fluviatilis*.

A total of 543 lampreys were genotyped at thirteen loci. All loci were in Hardy–Weinberg equilibrium and not impacted by null alleles for most populations, and there were no consistent issues for any given population (Table S4, Supporting information). A total of 112 of the 136 *F*_ST_ values (82.4%) were statistically significant (*P* < 0.05; Fig.3, Table S5, Supporting information). All *F*_ST_ values between *L. planeri* populations were significant with a range from 0.06045 to 0.191 (Wear vs. Nidd); however, only 45.4% of *F*_ST_ values for *L. fluviatilis* populations were significant, with a range from −0.00524 to 0.11945. When the freshwater-resident *L. fluviatilis* populations were not included, the *F*_ST_ values ranged from −0.00524 to 0.02537. *F*_ST_ values between *L. fluviatilis* and *L. planeri* populations ranged from 0.011 to 0.18554. Average allelic richness per locus ranged from 2.43 (Lp_003) to 14.9 (Lamper_4). Average *F*_IS_ per site ranged from −0.095 [Wear (*L. planeri*)] to 0.028 [Lomond (anadromous *L. fluviatilis*)].

**Fig 4:**
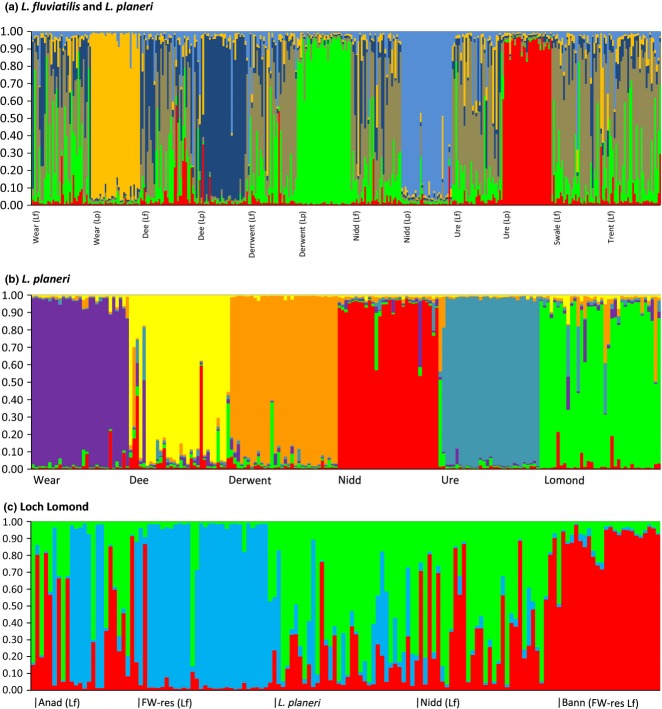
structure bar plot generated from microsatellite data for three population clusters of lampreys. (a) Comparison between *Lampetra fluviatilis* and *Lampetra planeri* populations (*K* = 6); (b) *L. planeri* populations (*K* = 6); (c) Loch Lomond populations compared to a population of *L. fluviatilis* from the Humber catchment and freshwater-resident *L. fluviatilis* populations from the R. Bann in N. Ireland (*K* = 3). Please see the open-access online paper for a colour version of this figure.

In COLONY, tests for the proportion of putative full-siblings (as an indicator of close kin) in populations of either species showed this to be rare, 0% in some cases for both species, and no higher than 0.74%. One randomly chosen individual of each full-sibling pair was excluded, and analysis was repeated. There were no differences that affected inference in the results with full-siblings included or excluded, so all individuals were included in the analysis.

structure analyses consistently identified *L. planeri* populations as being separate from *L. fluviatilis* populations (anadromous and freshwater-resident) and from each other (Fig.4). The only exception was the small sample of *L. planeri* on the Swale compared to the *L. fluviatilis* population downstream on the same river (Fig. S2a, Supporting information). Figure4a shows the most likely population structure among 12 sampling locations in England and Wales (excluding the Scottish Loch Lomond system) incorporating both species, where *K* = 6 showed the highest *LnP(D*) (Fig. S3, Supporting information). *Lampetra fluviatilis* samples appear as a single mixed population. Representing a higher hierarchical level, *ΔK* = 2 primarily supports separation of *L. fluviatilis* and *L. planeri* (Fig. S3 and S4, Supporting information). Figure S4c (Supporting information) shows a comparison across all populations where *K* = 9 [the highest *LnP*(D) outcome]. There were several peaks for *ΔK* at *K* = 2, 5 and 8 (Fig. S4d, Supporting information), but the maximum *LnP*(D) result (*K* = 9) was most informative, distinguishing all *L. planeri* and *L. fluviatilis* freshwater-resident populations (with the exception of the *L. planeri* population in the Swale).

When only *L. planeri* populations were compared, the highest likelihood result identified all populations as distinct (Fig.4b). In this case, *ΔK* was 4 (Fig. S3 and S4, Supporting information); however, this linked samples from the Nidd with the Dee, and Loch Lomond with the Derwent, in each case populations on opposite sides of British Isles (see Fig.1; Fig. S4b, Supporting information). When only anadromous *L. fluviatilis* populations were compared, the outcome was *K* = 1 (not shown). The Loch Lomond system (which contains anadromous *L. fluviatilis*, freshwater-resident *L. fluviatilis* and *L. planeri* populations) was compared to an anadromous *L. fluviatilis* population (Nidd) and another freshwater-resident *L. fluviatilis* population (Bann). structure identified three populations with highest likelihood, while *ΔK* was 2 (Fig.4c; Fig. S3, Supporting information). Using prior location information for Loch Lomond, five populations were identified. However, *ΔK* = 2, showing differentiation at a higher hierarchical level between the freshwater-resident *L. fluviatilis* population in Loch Lomond and the other populations (Fig. S2b,c, Supporting information). Location priors did not provide any useful additional inference for other analyses.

**Fig 5:**
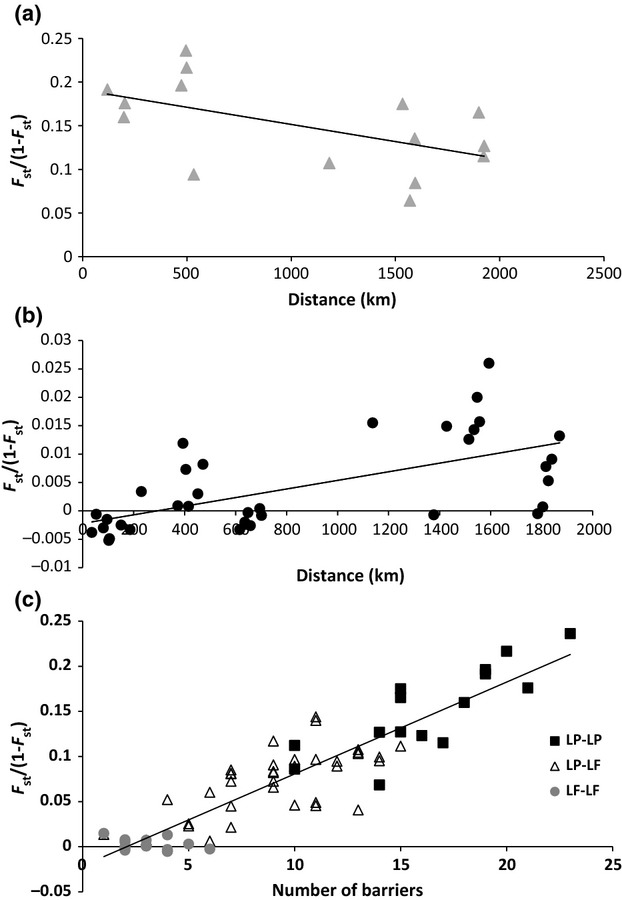
Isolation by distance tests for correlation between genetic differentiation (based on microsatellites) showing (a) geographic distance between freshwater-resident *Lampetra planeri* populations (*R*^2^ = 0.30, *P* < 0.05) and (b) geographic distance between anadromous *Lampetra fluviatilis* populations (*R*^2^ = 0.40, *P* < 0.0001; i.e. excluding freshwater-resident Bann and Lomond Lf). Inclusion of freshwater-resident Lf populations in the analysis reduced the strength of the correlation (*R*^2^ = 0.0841, *P* < 0.05)—not shown. (c) number of barriers between samples sites (*R*^2^ = 0.8256, *P* < 0.0001) where LP–LP signifies comparison of numbers of barriers between *L. planeri* sampling sites, LP–LF is number of barriers between *L. planeri* and *L. fluviatilis* sampling sites, and LF–LF is the number of barriers between *L. fluviatilis* sampling sites. Only sites for which barrier information was available were included in the analysis (i.e. Lf and Lp for Wear, Dee, Derwent, Nidd, Ure, and Swale Lf only).

The FCA plots support essentially the same clusters as identified in structure showing *L. fluviatilis* as being dominated by one large grouping, with the freshwater-resident populations differentiated (Fig. S5a, Supporting information) and *L. planeri* populations as all being separated from each other (Fig. S5b, Supporting information). Mantel tests for correlation between genetic and geographic distance showed a significant negative trend for *L. planeri* populations (*R*² = 0.2963; *P* < 0.05; Fig.5a) and a weak but significant positive linear relationship for all *L. fluviatilis* populations (*R*^2^ = 0.0841; *P* < 0.05). However, when freshwater-resident *L. fluviatilis* populations were excluded (Bann and Loch Lomond), the positive relationship was much stronger (*R*^2^ = 0.40, *P* < 0.0001; Fig.5b). Mantel tests examining correlations between genetic distance and the number of barriers along migration/dispersal routes for *L. fluviatilis* and *L. planeri* (populations included as described in methods) showed a highly significant positive correlation (*R*^2^ = 0.8256, *P* < 0.0001; Fig.5c).

Migration rate estimates between species (using migrate-n) ranged from 3.73 to 10.43 migrants/generation from *L. fluviatilis* to *L. planeri*, and from 4.18 to 16.28 from *L. planeri* to *L. fluviatilis* (Table S6, Supporting information). The six pairwise comparisons all suggested asymmetric gene flow greater in the direction from *L. planeri* to *L. fluviatilis* (which apart from Loch Lomond was always in the downstream direction), but 95% confidence intervals were large and overlapping. The bayesass analysis indicated low-level contemporary gene flow between the putative species, and some comparisons also suggest the downstream direction from *L. planeri* to *L. fluviatilis* (especially in the Derwent; Table S7a,b, Supporting information). It also indicated ongoing gene flow between the three forms in the Loch Lomond system (Table S7b, Supporting information).

## Discussion

### Population history

This study was based in a geographic region that has undergone profound cyclical changes over the course of the Pleistocene (2.58 Ma–11 700 years ago), with suitable riverine habitat available only during interglacial periods (Hays *et al*. 1976). For our study sites in the UK, mtDNA failed to show any differentiation between the two putative *Lampetra* species or among populations, which is consistent with data for some other northern European populations (Espanhol *et al*. 2007). While this may suggest ongoing gene flow or the incomplete sorting of ancestral polymorphisms, it is also consistent with recent founder events establishing these populations. The network analyses and neutrality tests support this, indicating small founder populations and subsequent expansion. Conversely, for populations in southern Europe where the climate has been more stable over time, there are far higher nucleotide diversities and significant mtDNA phylogeographic structuring (Pereira *et al*. 2010; Mateus *et al*. 2011).

Espanhol *et al*. (2007) suggested that *Lampetra planeri* in Europe may be polyphyletic and have originated within at least two evolutionary lineages, possibly the result of independent divergence events from *Lampetra fluviatilis* with the repeated loss of anadromy. Pereira *et al*. (2010) have since found several Portuguese populations of *L. planeri* which are isolated among themselves and also from the anadromous lamprey population. These populations had only private haplotypes, suggesting that a significant amount of time had passed to establish independent evolutionary histories. The fact that genetically distinct non-migratory *Lampetra* populations are found in many Portuguese rivers (Pereira *et al*. 2010; Mateus *et al*. 2011, 2013a) suggests lamprey were once more abundant and widespread in Iberia. The higher levels of divergence shown in our mtDNA median-joining network that included Portuguese lampreys (Fig.2), compared to other populations examined across Europe, also suggests that sufficient time may have passed to establish a complex of incipient freshwater-resident species, although further nuclear DNA data would help resolve this question. Similar processes generating multiple origins have been suggested, for example, in the marine to freshwater transitions of three-spine sticklebacks (*Gasterosteus aculeatus*; Hohenlohe *et al*. 2010).

Our study estimates the expansion time of *L. planeri* and *L. fluviatilis* populations in the British Isles and northern Europe as 16 236 (10 182–26 952) years ago using *tau* and a mutation rate of 5% per million years, which roughly coincides with the last glacial maximum (19–26 000 years ago; Clark *et al*. 2009). The Pleistocene climatic fluctuations impacted much of Europe (Hays *et al*. 1976; Webb & Bartlein 1992) and significantly influenced the distribution and genetic diversity of plants and animals (Hofreiter & Stewart 2009). In addition to cycles of habitat loss and release as glaciers extended and receded, the ‘refugium theory’ proposes that temperate species survived the glacial maxima in southern refugia and colonized northern latitudes during interglacial periods (Taberlet *et al*. 1998; Hewitt 2000). The results shown here, coupled with data from the Iberian Peninsula, suggest that southern latitudes served as an important refugium for *Lampetra* during the Pleistocene glaciations, intermittently acting as a point of dispersal for postglacial expansion (Espanhol *et al*. 2007; Mateus *et al*. 2012, 2013b).

Therefore, there may have been a tendency during interglacial periods, while anadromous *Lampetra* were expanding northwards, for populations at lower latitudes to abandon anadromy and eventually become restricted to freshwater. This is consistent with the findings of a recent study utilizing restriction site associated DNA sequencing (RAD seq.) that identified strong genetic differentiation between sympatric *L. fluviatilis* and *L. planeri* in the Iberian Peninsula with numerous fixed and diagnostic single nucleotide polymorphisms (SNPs) between the two putative species, some associated with genes related to osmoregulation (Mateus *et al*. 2013b). A study using RAD sequencing to compare Pacific lamprey (*Entosphenus tridentatus*) geographic populations also found evidence consistent with local adaptation (Hess *et al*. 2013). Our median-joining network in Fig.2 shows that for the available samples, only clade IV shares a haplotype with the lineage representing the northern expansion, suggesting a possible link between these lineages (with clade IV providing the ancestor of the anadromous group that founded the postglacial population in northern Europe). With expansion into previously unoccupied territory, it is expected that genetic diversity should decrease from the south to the north (Hewitt 2000), consistent with our findings.

### Population structure

In contrast to mtDNA, we found considerable structure at microsatellite DNA loci between *L. fluviatilis* and *L. planeri* populations, especially among populations of *L. planeri,* but much less among anadromous *L. fluviatilis* populations. Anadromous lampreys (*Lethenteron* spp.) in Japan (Yamazaki *et al*. 2011) and *Petromyzon mari-nus* in North America (Bryan *et al*. 2005) and Europe (Almada *et al*. 2008) exhibit similar levels of panmixia, with little or no genetic structure, despite their widespread distribution. Spice *et al*. (2012) found that Pacific lamprey along the west coast of North America showed low but significant differentiation among locations. However, instead of being philopatric like many other anadromous fish species (McDowall 2001), differentiation was suggested to be due to greater restrictions to dispersal at sea compared to other anadromous lamprey species. The lack of population structure found in our study was, therefore, consistent with the general lack of natal homing seen for other anadromous lamprey species.

The absence of a clear genetic signal for species-level differences between anadromous and freshwater-resident populations is consistent with findings for other paired lamprey species (Espanhol *et al*. 2007; Hubert *et al*. 2008; Docker 2009; April *et al*. 2011; Mateus *et al*. 2011; Boguski *et al*. 2012; Docker *et al*. 2012). Greater differentiation among populations within *L. planeri*, than between *L. planeri* and *L. fluviatilis,* suggests the unexpected pattern of greater gene flow between the putative species than within *L. planeri* (while the greatest gene flow occurs among populations of *L. fluviatilis*). Gene flow between the putative species may be possible owing to a combination of interspecific nest association (Huggins & Thompson 1970; Lasne *et al*. 2010) and sneaker male behaviour (Malmqvist 1983; Hume *et al*. 2013). As larvae of both species tend to move downstream through voluntary and involuntary drift behaviour (Hardisty & Potter 1971a; Moser *et al*. 2015), the distribution and overlap of spawning adults of the two species ultimately depends on a combination of the degree of downstream drift of *L. planeri* from upstream tributaries where they predominate, towards *L. fluviatilis*-dominated zones, and the subsequent upstream movements of freshwater-resident *L. planeri* and anadromous or freshwater-resident *L. fluviatilis* (Hardisty & Potter 1971b; Malmqvist 1980).

Both assignment (bayesass) and coalescent (migrate-n) methods suggested directionality in genetic migration, favouring the direction of *L. planeri* to *L. fluviatilis*, although the confidence limits were broad. Asymmetric gene flow occurring in these types of freshwater systems can significantly influence the distribution of genetic variation, with downstream populations typically exhibiting higher genetic diversity than headwater populations (Caldera & Bolnick 2008; Morrissey & de Kerckhove 2009; Julian *et al*. 2012). Yamazaki *et al*. (2011) found gene flow to exist at multitemporal scales between ‘potentially sympatric’ lamprey populations and suggested ongoing gene flow was the result of imperfect size-assortative mating and the plastic determination of life histories. The observed increase in genetic diversity as one moves downstream towards the lower reaches of the river could result from historical patterns of colonization, with contemporary dispersal reflecting movement bias, fragmented habitat or the presence of dispersal barriers (Morrissey & de Kerckhove 2009; Dehais *et al*. 2010). Asymmetric gene flow would be expected if *L. planeri* populations remain primarily resident further up the catchments with occasional migrants moving further downstream to where they may encounter spawning *L. fluviatilis*.

### Connectivity and anthropogenic factors

Mantel tests for isolation by distance revealed a positive correlation between geographic and genetic distance for anadromous *L. fluviatilis*, and a counterintuitive negative correlation among *L. planeri* populations (Fig.5). However, while the correlation for *L. fluviatilis* was significant (especially when freshwater-resident *L. fluviatilis* were omitted), and consistent with expectations (implying that long-range dispersal is less common), the correlation with *L. planeri* was weak and showed a broad range of values for a given distance (see Fig.5a). The *L. planeri* correlation may, therefore, simply reflect a stochastic pattern or ancestral relationships.

The number of anthropogenic barriers between populations was found to be significantly positively correlated with genetic distance, and such barriers have been shown to limit the upstream migration of *L. fluviatilis* (Lucas *et al*. 2009). Anthropogenic barriers could therefore be amplifying (beyond natural processes) the isolation of *L. planeri* populations by inhibiting the upstream movement of anadromous *L. fluviatilis* and preventing geneflow mediation in this manner between populations. Meldgaard *et al*. (2003) also detected a statistically significant increase of *F*_ST_ with the number of weirs between grayling (*Thymallus thymallus*) populations in a Danish river system. Similar decreases of genetic diversity from downstream towards upstream populations have been observed in other fish species in relation to anthropogenic barriers (Yamamoto *et al*. 2004; Caldera & Bolnick 2008; Raeymaekers *et al*. 2009). Yamazaki *et al*. (2011) found freshwater-resident nonparasitic lamprey populations in the upper regions of dammed rivers to be genetically divergent from seasonally sympatric, anadromous, parasitic populations. This pattern is consistent with a scenario where barriers amplify the asymmetry of gene flow from upstream towards downstream sites by allowing some passive downstream drift, while obstructing active upstream migration. Spice *et al*. (2012) also found that larvae from an anadromous population of *E. tridentatus* at a spawning site upstream of nine dams (which only a small number of adults successfully pass each year) exhibited higher genetic differentiation (i.e. higher *F*_ST_ values) than most other population comparisons.

When a freshwater-resident lamprey population is physically isolated from anadromous parasitic populations (which may mediate gene flow between freshwater-resident populations), acceleration in genetic divergence may result in the subsequent establishment of allopatric speciation (Yamazaki & Goto 2000). It is probable, however, that freshwater-resident *L. planeri* populations would have become, and tended to remain, isolated without the added anthropogenic hurdles, as there is a degree of population separation that is due to the natural extent of upstream migration in anadromous *L. fluviatilis*. As previous studies have shown, this is usually limited to higher order channels, and individuals do not generally penetrate the smaller streams even where access is unhindered by barriers (Hardisty & Potter 1971c; Hardisty 1986b).

The system in Loch Lomond offers evidence of the potential for gene flow between morphologically differentiated ecotypes, indicating that where they are found sympatrically, gene flow between *L. fluviatilis* and *L. planeri* can occur. This scenario is also supported by the lack of evidence for differentiation between the geographically proximate *L. fluviatilis* and *L. planeri* populations on the River Swale, although the sample size for the latter population was small (Fig. S2, Supporting information). Similarly, Docker *et al*. (2012) found no genetic differentiation between silver (*Ichthyomyzon unicuspis*) and northern brook (*I. fossor*) lampreys occurring sympatrically (also using microsatellite loci), but did find differentiation among parapatric populations. Yamazaki *et al*. (2011) also found a lack of differentiation between sympatric populations of Arctic lamprey (*Lethenteron camtschaticum*) and its nonparasitic derivatives in the Ohno River, Japan.

The bayesass analysis suggests that contemporary gene flow is occurring between all three populations in Loch Lomond, consistent with a tendency for interbreeding when there are no environmental barriers to limit connectivity. The divergence of the freshwater-resident *L. fluviatilis* population would then suggest a period of differentiation in isolation. Therefore, in Loch Lomond, the anadromous strategy is also paralleled by a population component with potamodromous behaviour, with some fish apparently showing migration mostly between the loch and spawning streams. While all three Loch Lomond populations were significantly differentiated from each other (Fig.3, Table S5, Supporting information), there were also data indicating contemporary gene flow among them, and the anadromous *L. fluviatilis* and sympatric *L. planeri* populations both showed evidence of connectivity with the wider *L. fluviatilis* populations.

## Conclusions

Alternative life history strategies are common among fishes inhabiting postglacial lakes, often resulting from adaptation to different foraging strategies or environments (Robinson & Parsons 2002). This is one of the best supported mechanisms for speciation in sympatry, for example among cichlid species in Holocene lakes (Barluenga *et al*. 2006). The divergence of multiple independent populations is a common trend in the evolution of diversity for diadromous fish (Schluter & Nagel 1995; Waters & Wallis 2001), and a number of studies have shown the influence of glacial movement within the Holocene on the phylogeographical structure of freshwater fishes (Harris & Taylor 2010; Boguski *et al*. 2012). However, in our study, the geographic scale is small for the extent of differentiation observed. It is apparent that at an initial stage, there was a postglacial expansion of anadromous *Lampetra fluviatilis* from southern refugia and the subsequent establishment of multiple freshwater-resident *Lampetra planeri* populations. These may have been relatively small founder groups that retained some degree of reproductive isolation that was likely intensified, although perhaps not entirely determined, by the anthropogenic introduction of barriers. Moreover, it was ascertained that there is gene flow between *L. fluviatilis* and *L. planeri* in both long-term and contemporary timescales and the pattern of gene flow is apparently asymmetric. This has significant implications for the management of *L. planeri* populations and the extent to which this is underpinned by natural processes will have important evolutionary implications with respect to the mechanisms that generate diversity. Our data emphasizes the importance of founder events in the evolution of diversity among populations and as a frequent component of the speciation process (Templeton 2008). These data also strongly support a scenario of multitemporal and multispatial radiation. In contrast to higher levels of *Lampetra* divergence present in the Iberian Peninsula, the northern European populations appear to have been established relatively recently, and the process of differentiation is still ongoing. There may be a natural tendency towards speciation in freshwater-resident populations that remain environmentally stable over time, but a dynamic process instead at higher latitudes experiencing a cycle of habitat loss and release.

## Data accessibility

DNA sequences: GenBank accessions: KP722176–KP722191; microsatellite DNA genotypes and mtDNA sequence alignment file: Dryad doi:10.5061/dryad.v105s.

## Supporting information

**Table S1** Numbers collected and location of origin for all genetic samples of *Lampetra planeri* and *Lampetra fluviatilis* and by whom they were collected. **Table S2** Number of barriers (distance, km) between sampling locations. **Table S3** Details of microsatellite loci and primers utilised in the study. **Table S4** Table outlining diversity indices for microsatellite loci by population, and by locus. **Table S5** Pairwise *F*_ST_ values for 13 microsatellite loci. **Table S6** migrate-n analysis (Bayesian) showing posterior distributions for six paired sites (95% confidence intervals in brackets). **Table S7** Pairwise estimation of current gene flow, *M*, between (a) populations of anadromous *Lampetra fluviatilis* (Lf) and *Lampetra planeri* (Lp) from within the same river, and (b) populations within Loch Lomond by bayesass. **Fig. S1** Mismatch distribution (demographic expansion) with *Tau* 0.673, showing an expansion pattern for the six populations of *Lampetra fluviatilis* and *Lampetra planeri* presented in Table 1. **Fig. S2** structure bar plot generated from microsatellite data showing (a) *ΔK* = 2 (Ln*P*(*D*) *=* −2294.8) where Swale Lp is compared to another *Lampetra planeri* population and *Lampetra fluviatilis* form the same river (b) *ΔK* = 2 when prior location information is used to analyse the Loch Lomond system which shows the freshwater-resident Lomond population to be differentiated and (c) *ΔK* = 5 (Ln*P*(*D*) *=* −4681) when a location prior is used. **Fig. S3** Posterior probability of the data (Ln[*P*(*D*|*K*)]) and values of *ΔK* (Evanno *et al*. 2005) as a function of *K* (number of clusters), associated with the results shown in Fig. 4 in the main text. **Fig. S4** (a) Structure for 12 populations where *ΔK* = 2. (b) Structure for *Lampetra planeri* only where *ΔK* = 4. (c) Structure for all samples included when *K* = 10 (Lf = *Lampetra fluviatilis*, Lp = *L. planeri*, LL = Loch Lomond; anad = anadromous; res = resident). (d) *ΔK* plot for the structure plot in part c. **Fig. S5** FCA analysis for (a) *Lampetra fluviatilis* and (b) *Lampetra planeri* population.
