# Supplementary material for: Contrasting population genetic structure among freshwater-resident and anadromous lampreys: the role of demographic history, differential dispersal and anthropogenic barriers to movement
Source: Mol Ecol. 2015 Mar 6;24(6):1188–204. doi: 10.1111/mec.13112 (PMC4413359; doi:10.1111/mec.13112)
Supplement: Supplementary file 1 [file mec0024-1188-sd1.docx]

**Table S1**. Numbers collected and location of origin for all genetic samples of *L. planeri* and *L. fluviatilis* and by whom they were collected. Sites 1-8 are all part of the Ouse subcatchment within the Humber, while site 9 is in the Trent subcatchment (Fig 1). The seven systems where anadromous *L. fluviatilis* and freshwater *L. planeri* occur in close proximity (i.e. seven paired sites) are outlined in boxes and given a paired site number. * signify sites used for MtDNA analysis.

| Site no. On  Map | Paired Site No. | Country | Catchment | River | Latitude | Longitude | Species | *N* | Method of collection | Collected by: |
| --- | --- | --- | --- | --- | --- | --- | --- | --- | --- | --- |
| 1* | **PS1** | England | Humber | Nidd | 53°58'49.00"N | 1°19'5.99" W | *L. fluviatilis* | 30 | Hand net | F. Bracken |
| 2* |  | England | Humber | Nidd | 54° 4'38.33" N | 1°44'48.81" W | *L. planeri* | 30 | Hand net | F. Bracken |
| 3 | **PS2** | England | Humber | Swale | 54°21'28.20" N | 1°32'59.70" W | *L. fluviatilis* | 32 | Hand net | F. Bracken |
| 4 |  | England | Humber | Skeeby Beck (Swale) | 54°25'16.14" N | 1°41'14.29" W | *L. planeri* | 11 | Electro-fishing | F. Bracken |
| 5 | **PS3** | England | Humber | Ure | 54° 5'50.94" N | 1°23'44.63" W | *L. fluviatilis* | 30 | Hand net | F. Bracken |
| 6 |  | England | Humber | Burn (Ure) | 54°13'1.92" N | 1°43'27.06" W | *L. planeri* | 30 | Electro-fishing | F. Bracken |
| 7 | **PS4** | England | Humber | Derwent | 53°59'31.06" N | 0°54'50.49" W | *L. fluviatilis* | 30 | Hand net | F. Bracken |
| 8 |  | England | Humber | Rye (Derwent) | 54°14'13.29" N | 1° 2'32.93" W | *L. planeri* | 32 | Hand net | F. Bracken |
| 9 |  | England | Humber | Trent | 53° 8'41.27" N | 0°47'28.42" W | *L. fluviatilis* | 33 | Trap | F. Bracken & P. Bird |
| 10 | **PS5** | England | Wear | Wear | 54°46'49.31" N | 1°34'34.85" W | *L. fluviatilis* | 43 | Hand net | F. Bracken & M. Lucas |
| 11 |  | England | Wear | Bollihope Burn | 54°43'19.28" N | 1°56'37.19" W | *L. planeri* | 30 | Electro-fishing | F. Bracken & M. Lucas |
| 12* | **PS6** | Wales | Dee | Dee | 53°11'11.37"N | 2°53'14.43"W | *L. fluviatilis* | 33 | Trap and electro-fishing | I. Davidson, R. Cove & F. Bracken |
| 13* |  | Wales | Dee | Ceiriog | 52°55'35.29" N | 3° 4'58.55" W | *L. planeri* | 30 | Electro-fishing | F. Bracken |
| 14 | **PS7** | Scotland | Loch Lomond | Endrick Water | 56° 3'17.31"N | 4°27'16.28"W | *L. fluviatilis* (Anadromous) | 24 | Trap | J. Hume |
| 15 |  | Scotland | Loch Lomond | Endrick Water | 56° 3'17.31"N | 4°27'16.28"W | *L. fluviatilis* (Resident) | 31 | Trap | J. Hume |
| 16 |  | Scotland | Loch Lomond | Endrick Water | 56° 3'17.31"N | 4°27'16.28"W | *L. planeri* | 36 | Trap | J. Hume |
| 17* |  | N. Ireland | Bann | Bann | 54°45'18.69" N | 6°27'51.06" W | *L. fluviatilis* (Resident) | 25 | Trap | Clare Goodwin |
| 18* |  | Belgium | Scheldt | Scheldt | 51° 0'25.89" N | 3°45'7.89" E | *L. fluviatilis* | 35 | Trap | David Buysse & Johan Coeck |
|  |  |  |  |  |  |  | Total | 543 |  |  |

Site numbers correspond to those in Figure 1.

| **Derwent (Lp)** | **7** (54.6) |  |  |  |  |  |  |  |  |  | LP-LP |
| --- | --- | --- | --- | --- | --- | --- | --- | --- | --- | --- | --- |
| **Nidd (Lf)** | **4** (102.6) | **11** (157.2) |  |  |  |  |  |  |  |  | LP-LF |
| **Nidd (Lp)** | **14** (147.7) | **21** (202.3) | **10** (45.1) |  |  |  |  |  |  |  | LF-LF |
| **Ure (Lf)** | **4** (105.45) | **11** (160.05) | **2** (36.08) | **12** (81.8) |  |  |  |  |  |  |  |
| **Ure (Lp)** | **11** (143.25) | **18** (197.85) | **9** (73.88) | **19** (118.98) | **7** (37.8) |  |  |  |  |  |  |
| **Swale (Lf)** | **6** (150.85) | **13** (205.45) | **4** (81.4) | **14** (126.5) | **2** (53.5) | **9** (91.3) |  |  |  |  |  |
| **Swale (Lp)** | **7** (167.25) | **14** (221.85) | **5** (97.8) | **15** (142.9) | **3** (69.9) | **10** (107.7) | **1** (16.4) |  |  |  |  |
| **Wear (Lf)** | **3** (373.4) | **10** (428) | **3** (404.9) | **13** (450) | **3** (415.4) | **10** (453.2) | **5** (452) | **6** (468.43) |  |  |  |
| **Wear (Lp)** | **11** (419.1) | **19** (473.7) | **13** (450.6) | **23** (495.7) | **13** (461.1) | **20** (498.9) | **15** (497.7) | **16** (514.13) | **9** (45.7) |  |  |
| **Dee (Lf)** | **2** (1784.6) | **9** (1839.2) | **2** (1817.1) | **12** (1862.2) | **2** (1826.6) | **9** (1864.4) | **4** (1870.46) | **5** (1886.86) | **1** (1427.7) | **11** (1473.4) |  |
| **Dee (Lp)** | **7** (1845.7) | **15** (1900.3) | **7** (1878.2) | **17** (1923.3) | **7** (1887.7) | **14** (1925.5) | **9** (1931.56) | **10** (1947.96) | **6** (1488.8) | **15** (1534.5) | **4** (61.1) |
|  | **Derwent (Lf)** | **Derwent (Lp)** | **Nidd (Lf)** | **Nidd (Lp)** | **Ure (Lf)** | **Ure (Lp)** | **Swale (Lf)** | **Swale (Lp)** | **Wear (Lf)** | **Wear (Lp)** | **Dee (Lf)** |

**Table S2** Number of barriers (distance, km) between sampling locations. Boxes outlined with border signify paired sites. Lp = *Lampetra planeri*, Lf = *Lampetra fluviatilis.*  Blue boxes represent barriers between *L. planeri* populations, red boxes signify barriers between *L. planeri* and *L. fluviatilis* populations, and green boxes represent barriers between *L. fluviatilis* populations. Geographic distances were calculated between sample sites (based on the shortest possible route) using linear referencing tools in Quantum GIS (Lisboa). A barrier was as any feature larger than 0.5 m height at base river level which reaches the full width of the river.

**Table S3.** Details of microsatellite loci and primers utilised in the study.

Loci and primer sequences

| Locus | Primer Sequence (5'–3') | Reference | Allele Size(bp) |
| --- | --- | --- | --- |
| LP-003 | F: TCACGTACGCGTTAACTCCA | (Gaigher *et al.*, 2013) | 88-96 |
|  | R: TTCCTTAATTGGTCTGCCTCAGGA |  |  |
| LP-009 | F: AACTCCCACGTGCAAAATTC | (Gaigher *et al.*, 2013) | 188-194 |
|  | R: AGGCATCACTCCTAACGACG |  |  |
| Lamper_1 | F:GCGAGTGTCCGAGCAGCG | Developed by Author | 239-269 |
|  | R:TGCGGCACAACCGCGGAC |  |  |
| Lamper_2 | F:TTACAAGCCACCTTCTCC | Developed by Author | 154-410 |
|  | R: GCTGATGTTGGCAGTGAG |  |  |
| Lri-5 | F: GCCGACAACAACCAACATC | (Luzier *et al.,* 2010) | 257-278 |
|  | R: CACGCAGGTCACCCTCTAC |  |  |
| LP-027 | F: ACAGTCAACCTCCGACATCC | (Gaigher *et al.*, 2013) | 196-208 |
|  | R: AGCCCATGATGATTCCATTC |  |  |
| LP-028 | F: AGAACTCTGTGGACGTTCCG | (Gaigher *et al.*, 2013) | 231-243 |
|  | R: TCTCAAGAAATGAGTTCTCAATCG |  |  |
| Lp-046 | F: ACCGCAAACTCATCAGGAAC | (Gaigher *et al.*, 2013) | 134-146 |
|  | R: AAGCGGATTTAGAAGCGACA |  |  |
| Lamper_3 | F: TGAGGGTCCTGGTGTGCG | Developed by Author | 208-228 |
|  | R: GGCAGGAGGTCACGGGC |  |  |
| LP-006 | F: TGCCCACACGTGATAGACAT | (Gaigher *et al.*, 2013) | 114-134 |
|  | R: GGCGATCGTCATAAATAGCC |  |  |
| LP-045 | F: AGAGGTGTTTCGCGTGCTAT | (Gaigher *et al.*, 2013) | 170-185 |
|  | R: AAGGAGAGAGGAGGTTTCGG |  |  |
| LP-018 | F: TTAAAAGTGCGGCGAAATCT | (Gaigher *et al.*, 2013) | 224-238 |
|  | R: TGTTCCATAACCACTGCTCG |  |  |
| Lamper_4 | F: TACCCCGTGGCACTTGAC | Developed by Author | 254-542 |
|  | R: TGGACGCCGGAGTTGACC |  |  |

**Table S4.** Table outlining diversity indices for microsatellite loci by population, and by locus. Where Lp = *L. planeri*, Lf = *L. fluviatilis,* and Lf Res = freshwater resident population of *L. fluviatilis. Na* = number of alleles, *Ar* = allelic richness, *Ho* = observed homozygosity, *He* = expected homozygosity, *p-value* =indicates significance of deviation from Hardy-Weinberg equilibrium (after Bonferroni correction significance level was adjust to *P <* 0.000226. Any P-values still significant after this are highlighted in a grey box). *Fis* = inbreeding coefficient and *Null alleles =* presence of any null alleles which are highlighted in a green box and shown as estimated allele frequency using the Oosterhout algorithm.

|  | **Locus** | **Lp_003** | **Lp_009** | **Lamper_1** | **Lamper _2** | **Lri_5** | **Lp_027** | **Lp_028** | **Lp_046** | **Lamper _3** | **Lp_006** | **Lp_045** | **lp_018** | **Lamper_4** | **All** |
| --- | --- | --- | --- | --- | --- | --- | --- | --- | --- | --- | --- | --- | --- | --- | --- |
| **Wear (Lf)** | ***Na*** | 2.000 | 4.000 | 12.000 | 18.000 | 3.000 | 4.000 | 5.000 | 3.000 | 4.000 | 2.000 | 4.000 | 5.000 | 17.000 |  |
|  | ***Ar*** | 1.998 | 3.204 | 7.859 | 10.872 | 2.849 | 3.863 | 4.413 | 2.987 | 2.826 | 1.898 | 3.520 | 4.557 | 11.353 |  |
|  | ***Ho*** | 0.314 | 0.639 | 0.833 | 0.829 | 0.500 | 0.735 | 0.806 | 0.583 | 0.583 | 0.111 | 0.583 | 0.778 | 0.861 |  |
|  | ***He*** | 0.342 | 0.593 | 0.803 | 0.909 | 0.542 | 0.683 | 0.683 | 0.521 | 0.504 | 0.155 | 0.597 | 0.665 | 0.921 |  |
|  | ***p-value*** | 0.631 | 0.193 | 0.512 | 0.034 | 0.156 | 0.947 | 0.384 | 0.887 | 0.281 | 0.203 | 0.925 | 0.030 | 0.282 |  |
|  | ***Fis*** | 0.081 | -0.079 | -0.039 | 0.090 | 0.078 | -0.077 | -0.182 | -0.122 | -0.159 | 0.286 | 0.023 | -0.173 | 0.066 | -0.040 |
|  | ***Null alleles*** |  |  |  |  |  |  |  |  |  |  |  |  |  |  |
| **Wear (Lp)** | ***Na*** | 2.000 | 3.000 | 2.000 | 11.000 | 3.000 | 3.000 | 3.000 | 2.000 | 2.000 | 2.000 | 2.000 | 3.000 | 7.000 |  |
|  | ***Ar*** | 2.000 | 2.995 | 1.619 | 7.979 | 2.374 | 2.379 | 2.480 | 2.000 | 2.000 | 1.769 | 2.000 | 2.619 | 6.117 |  |
|  | ***Ho*** | 0.621 | 0.586 | 0.069 | 0.724 | 0.379 | 0.448 | 0.207 | 0.448 | 0.310 | 0.103 | 0.731 | 0.724 | 0.724 |  |
|  | ***He*** | 0.508 | 0.638 | 0.068 | 0.789 | 0.319 | 0.399 | 0.194 | 0.390 | 0.390 | 0.100 | 0.509 | 0.541 | 0.742 |  |
|  | ***p-value*** | 0.278 | 0.025 | 1.000 | 0.622 | 0.631 | 0.730 | 1.000 | 0.636 | 0.338 | 1.000 | 0.046 | 0.046 | 0.541 |  |
|  | ***Fis*** | -0.226 | 0.083 | -0.018 | 0.084 | -0.194 | -0.127 | -0.070 | -0.152 | 0.208 | -0.037 | -0.448 | -0.346 | 0.025 | -0.095 |
|  | ***Null alleles*** |  |  |  |  |  |  |  |  |  |  |  |  |  |  |
| **Dee (Lf)** | ***Na*** | 3.000 | 4.000 | 10.000 | 21.000 | 3.000 | 5.000 | 4.000 | 3.000 | 4.000 | 2.000 | 5.000 | 5.000 | 24.000 |  |
|  | ***Ar*** | 2.557 | 3.539 | 6.864 | 11.688 | 2.811 | 4.317 | 3.969 | 2.993 | 2.892 | 1.878 | 3.608 | 4.148 | 12.345 |  |
|  | ***Ho*** | 0.455 | 0.515 | 0.727 | 0.818 | 0.364 | 0.607 | 0.727 | 0.697 | 0.515 | 0.152 | 0.419 | 0.727 | 0.909 |  |
|  | ***He*** | 0.386 | 0.570 | 0.748 | 0.897 | 0.459 | 0.687 | 0.737 | 0.615 | 0.551 | 0.142 | 0.599 | 0.574 | 0.916 |  |
|  | ***p-value*** | 0.173 | 0.517 | 0.207 | 0.553 | 0.257 | 0.339 | 0.014 | 0.662 | 0.863 | 1.000 | 0.136 | 0.429 | 0.184 |  |
|  | ***Fis*** | -0.181 | 0.098 | 0.028 | 0.090 | 0.210 | 0.118 | 0.013 | -0.136 | 0.065 | -0.067 | 0.303 | -0.273 | 0.008 | 0.013 |
|  | ***Null alleles*** |  |  |  |  |  |  |  |  |  |  | 0.135 |  |  |  |
| **Dee (Lp)** | ***Na*** | 2.000 | 4.000 | 9.000 | 8.000 | 3.000 | 4.000 | 4.000 | 3.000 | 2.000 | 3.000 | 3.000 | 3.000 | 12.000 |  |
|  | ***Ar*** | 1.994 | 3.323 | 5.977 | 5.710 | 2.999 | 3.970 | 3.748 | 2.698 | 2.000 | 1.969 | 2.965 | 2.363 | 8.030 |  |
|  | ***Ho*** | 0.267 | 0.533 | 0.700 | 0.714 | 0.700 | 0.667 | 0.600 | 0.300 | 0.367 | 0.100 | 0.556 | 0.400 | 0.733 |  |
|  | ***He*** | 0.282 | 0.458 | 0.714 | 0.686 | 0.654 | 0.674 | 0.613 | 0.269 | 0.503 | 0.098 | 0.570 | 0.332 | 0.840 |  |
|  | ***p-value***  Cont. | 1.000 | 1..000 | 0.233 | 0.292 | 1.000 | 0.393 | 0.766 | 1.000 | 0.159 | 1.000 | 1.000 | 0.633 | 0.010 |  |
|  | ***Fis*** | 0.057 | -0.169 | 0.020 | -0.042 | -0.072 | 0.012 | 0.022 | -0.115 | 0.275 | -0.024 | 0.025 | -0.210 | 0.128 | -0.016 |
|  | ***Null alleles*** |  |  |  |  |  |  |  |  |  |  |  |  |  |  |
| **Derwent (Lf)** | ***Na*** | 3.000 | 4.000 | 9.000 | 19.000 | 4.000 | 4.000 | 5.000 | 3.000 | 5.000 | 3.000 | 4.000 | 6.000 | 20.000 |  |
|  | ***Ar*** | 2.617 | 3.297 | 6.661 | 12.413 | 3.297 | 3.982 | 4.385 | 2.998 | 3.336 | 2.215 | 3.372 | 4.657 | 12.018 |  |
|  | ***Ho*** | 0.448 | 0.586 | 0.586 | 0.846 | 0.483 | 0.852 | 0.786 | 0.567 | 0.567 | 0.167 | 0.519 | 0.607 | 0.800 |  |
|  | ***He*** | 0.387 | 0.595 | 0.717 | 0.932 | 0.528 | 0.679 | 0.760 | 0.631 | 0.570 | 0.158 | 0.591 | 0.718 | 0.920 |  |
|  | ***p-value*** | 0.294 | 0.152 | 0.039 | 0.035 | 0.752 | 0.588 | 0.963 | 0.736 | 0.445 | 1.000 | 0.240 | 0.535 | 0.104 |  |
|  | ***Fis*** | -0.161 | 0.016 | 0.185 | 0.094 | 0.086 | -0.262 | -0.035 | 0.103 | 0.006 | -0.058 | 0.124 | 0.157 | 0.132 | -0.004 |
|  | ***Null alleles*** |  |  |  |  |  |  |  |  |  |  |  |  | 0.058 |  |
| **Derwent (Lp)** | ***Na*** | 4.000 | 3.000 | 6.000 | 11.000 | 2.000 | 3.000 | 4.000 | 4.000 | 3.000 | 3.000 | 3.000 | 4.000 | 15.000 |  |
|  | ***Ar*** | 2.687 | 2.724 | 4.012 | 7.066 | 2.000 | 2.999 | 3.983 | 3.343 | 2.344 | 2.068 | 2.753 | 2.687 | 10.417 |  |
|  | ***Ho*** | 0.500 | 0.531 | 0.656 | 0.781 | 0.387 | 0.767 | 0.750 | 0.656 | 0.375 | 0.125 | 0.533 | 0.625 | 1.000 |  |
|  | ***He*** | 0.479 | 0.487 | 0.549 | 0.814 | 0.503 | 0.658 | 0.738 | 0.670 | 0.469 | 0.121 | 0.481 | 0.479 | 0.909 |  |
|  | ***p-value*** | 1.000 | 0.862 | 0.126 | 0.051 | 0.279 | 0.293 | 0.135 | 0.513 | 0.391 | 1.000 | 0.727 | 0.181 | **0.000** |  |
|  | ***Fis*** | -0.045 | -0.093 | -0.200 | 0.041 | 0.234 | -0.169 | -0.017 | 0.020 | 0.203 | -0.038 | -0.111 | -0.312 | -0.102 | -0.058 |
|  | ***Null alleles*** |  |  |  |  |  |  |  |  |  |  |  |  |  |  |
| **Nidd (Lf)** | ***Na*** | 3.000 | 4.000 | 9.000 | 21.000 | 4.000 | 5.000 | 4.000 | 4.000 | 4.000 | 2.000 | 3.000 | 4.000 | 20.000 |  |
|  | ***Ar*** | 2.367 | 3.742 | 6.481 | 13.072 | 3.120 | 4.338 | 3.912 | 3.599 | 3.120 | 1.967 | 2.908 | 3.948 | 12.691 |  |
|  | ***Ho*** | 0.533 | 0.533 | 0.700 | 0.867 | 0.600 | 0.800 | 0.800 | 0.667 | 0.367 | 0.167 | 0.633 | 0.700 | 0.900 |  |
|  | ***He*** | 0.437 | 0.666 | 0.695 | 0.941 | 0.490 | 0.682 | 0.669 | 0.663 | 0.571 | 0.210 | 0.559 | 0.688 | 0.934 |  |
|  | ***p-value*** | 0.553 | 0.048 | 0.161 | 0.007 | 0.192 | 0.627 | 0.306 | 0.883 | 0.065 | 0.326 | 0.433 | 0.027 | 0.095 |  |
|  | ***Fis*** | -0.226 | 0.201 | -0.007 | 0.080 | -0.230 | -0.177 | -0.199 | -0.006 | 0.362 | 0.208 | -0.136 | -0.018 | 0.037 | -0.008 |
|  | ***Null alleles*** |  |  |  |  |  |  |  |  | 0.170 |  |  |  |  |  |
| **Nidd (Lp)** | ***Na*** | 2.000 | 4.000 | 2.000 | 8.000 | 2.000 | 4.000 | 4.000 | 3.000 | 2.000 | 1.000 | 4.000 | 4.000 | 7.000 |  |
|  | ***Ar*** | 2.000 | 3.301 | 1.999 | 6.033 | 2.000 | 3.215 | 3.312 | 2.989 | 2.000 | 1.000 | 3.450 | 3.829 | 5.057 |  |
|  | ***Ho*** | 0.867 | 0.367 | 0.433 | 0.655 | 0.367 | 0.500 | 0.633 | 0.533 | 0.467 | mono | 0.533 | 0.733 | 0.700 |  |
|  | ***He*** | 0.499 | 0.325 | 0.345 | 0.649 | 0.494 | 0.581 | 0.606 | 0.595 | 0.452 | mono | 0.489 | 0.633 | 0.699 |  |
|  | ***p-value*** | **0.000** | 1.000 | 0.291 | 0.410 | 0.255 | 0.613 | 0.360 | 0.735 | 1.000 | mono | 0.399 | 0.525 | 0.382 |  |
|  | ***Fis***  Cont. | -0.758 | -0.131 | -0.261 | -0.009 | 0.262 | 0.142 | -0.046 | 0.105 | -0.033 | N.A. | -0.093 | -0.162 | -0.001 | -0.071 |
|  | ***Null alleles*** |  |  |  |  |  |  |  |  |  |  |  |  |  |  |
| **Ure (Lf)** | ***Na*** | 3.000 | 3.000 | 8.000 | 24.000 | 2.000 | 4.000 | 4.000 | 3.000 | 4.000 | 2.000 | 3.000 | 5.000 | 23.000 |  |
|  | ***Ar*** | 2.379 | 2.995 | 6.136 | 13.917 | 2.000 | 3.981 | 3.936 | 2.984 | 2.759 | 1.984 | 2.918 | 4.582 | 13.426 |  |
|  | ***Ho*** | 0.586 | 0.621 | 0.759 | 0.966 | 0.276 | 0.793 | 0.586 | 0.586 | 0.552 | 0.276 | 0.552 | 0.828 | 0.828 |  |
|  | ***He*** | 0.482 | 0.617 | 0.733 | 0.942 | 0.436 | 0.724 | 0.673 | 0.615 | 0.528 | 0.242 | 0.578 | 0.675 | 0.938 |  |
|  | ***p-value*** | 0.498 | 0.307 | 0.721 | 0.822 | 0.077 | 0.955 | 0.558 | 0.168 | 0.810 | 1.000 | 0.409 | 0.056 | 0.083 |  |
|  | ***Fis*** | -0.221 | -0.006 | -0.035 | -0.026 | 0.371 | -0.097 | 0.131 | 0.047 | -0.047 | -0.143 | 0.046 | -0.232 | 0.120 | -0.003 |
|  | ***Null alleles*** |  |  |  |  |  |  |  |  |  |  |  |  | 0.053 |  |
| **Ure (Lp)** | ***Na*** | 2.000 | 3.000 | 4.000 | 10.000 | 2.000 | 3.000 | 3.000 | 4.000 | 3.000 | 2.000 | 3.000 | 4.000 | 8.000 |  |
|  | ***Ar*** | 2.000 | 2.847 | 3.734 | 6.968 | 2.000 | 3.000 | 2.845 | 3.355 | 2.967 | 1.849 | 2.603 | 3.328 | 5.324 |  |
|  | ***Ho*** | 0.600 | 0.467 | 0.533 | 0.867 | 0.367 | 0.500 | 0.500 | 0.600 | 0.667 | 0.133 | 0.500 | 0.433 | 0.467 |  |
|  | ***He*** | 0.472 | 0.425 | 0.640 | 0.835 | 0.481 | 0.678 | 0.406 | 0.572 | 0.605 | 0.127 | 0.520 | 0.458 | 0.595 |  |
|  | ***p-value*** | 0.233 | 0.825 | 0.001 | 0.898 | 0.250 | 0.027 | 0.532 | 0.900 | 0.143 | 1.000 | 0.030 | 0.235 | 0.039 |  |
|  | ***Fis*** | -0.276 | -0.100 | 0.169 | -0.039 | 0.240 | 0.266 | -0.236 | -0.049 | -0.104 | -0.055 | 0.040 | 0.055 | 0.218 | 0.027 |
|  | ***Null alleles*** |  |  |  |  |  |  |  |  |  |  |  |  |  |  |
| **Swale (Lf)** | ***Na*** | 4.000 | 3.000 | 8.000 | 27.000 | 4.000 | 5.000 | 5.000 | 4.000 | 4.000 | 4.000 | 4.000 | 6.000 | 20.000 |  |
|  | ***Ar*** | 2.687 | 2.984 | 6.201 | 14.325 | 2.687 | 4.297 | 4.299 | 3.338 | 2.687 | 2.890 | 3.529 | 4.574 | 12.466 |  |
|  | ***Ho*** | 0.438 | 0.688 | 0.750 | 0.906 | 0.406 | 0.750 | 0.563 | 0.594 | 0.750 | 0.344 | 0.563 | 0.688 | 0.844 |  |
|  | ***He*** | 0.412 | 0.615 | 0.697 | 0.949 | 0.441 | 0.727 | 0.668 | 0.601 | 0.531 | 0.302 | 0.607 | 0.676 | 0.931 |  |
|  | ***p-value*** | 0.519 | 0.700 | 0.378 | 0.116 | 0.822 | 0.057 | 0.072 | 0.566 | 0.013 | 1.000 | 0.556 | 0.082 | 0.158 |  |
|  | ***Fis*** | -0.064 | -0.121 | -0.077 | 0.046 | 0.081 | -0.032 | 0.160 | 0.012 | -0.423 | -0.140 | 0.074 | -0.018 | 0.095 | -0.016 |
|  | ***Null alleles*** |  |  |  |  |  |  |  |  |  |  |  |  |  |  |
| **Lomond (Lf)** | ***Na*** | 2.000 | 4.000 | 7.000 | 16.000 | 3.000 | 4.000 | 5.000 | 3.000 | 4.000 | 4.000 | 3.000 | 4.000 | 17.000 |  |
|  | ***Ar*** | 1.996 | 3.693 | 5.602 | 11.520 | 2.998 | 3.870 | 4.471 | 2.961 | 2.957 | 2.908 | 2.998 | 3.467 | 12.135 |  |
|  | ***Ho*** | 0.333 | 0.583 | 0.750 | 0.833 | 0.583 | 0.609 | 0.696 | 0.458 | 0.478 | 0.375 | 0.500 | 0.565 | 0.875 |  |
|  | ***He*** | 0.284 | 0.577 | 0.731 | 0.919 | 0.621 | 0.623 | 0.750 | 0.483 | 0.519 | 0.324 | 0.637 | 0.557 | 0.932 |  |
|  | ***p-value*** | 1.000 | 0.383 | 0.676 | 0.003 | 0.028 | 0.483 | 0.034 | 0.740 | 0.669 | 1.000 | 0.123 | 0.695 | 0.139 |  |
|  | ***Fis*** | -0.179 | -0.011 | -0.026 | 0.095 | 0.063 | 0.024 | 0.074 | 0.052 | 0.080 | -0.160 | 0.219 | -0.014 | 0.062 | 0.028 |
|  | ***Null alleles***  Cont. |  |  |  |  |  |  |  |  |  |  |  |  |  |  |
| **Lomond (Lf Res)** | ***Na*** | 2.000 | 3.000 | 5.000 | 11.000 | 3.000 | 5.000 | 2.000 | 4.000 | 3.000 | 3.000 | 3.000 | 5.000 | 10.000 |  |
|  | ***Ar*** | 2.000 | 2.836 | 4.191 | 8.091 | 2.739 | 4.550 | 2.000 | 2.942 | 2.355 | 2.900 | 2.977 | 3.777 | 7.472 |  |
|  | ***Ho*** | 0.677 | 0.710 | 0.774 | 0.667 | 0.710 | 0.677 | 0.226 | 0.645 | 0.581 | 0.419 | 0.484 | 0.613 | 0.742 |  |
|  | ***He*** | 0.455 | 0.565 | 0.722 | 0.829 | 0.554 | 0.694 | 0.373 | 0.517 | 0.428 | 0.359 | 0.538 | 0.500 | 0.848 |  |
|  | ***p-value*** | 0.006 | 0.014 | 0.838 | 0.008 | 0.003 | 0.027 | 0.042 | 0.140 | 0.087 | 0.775 | 0.577 | 0.135 | **0.000** |  |
|  | ***Fis*** | -0.500 | -0.261 | -0.074 | 0.199 | -0.288 | 0.025 | 0.398 | -0.254 | -0.365 | -0.171 | 0.102 | -0.231 | 0.127 | -0.080 |
|  | ***Null alleles*** |  |  |  |  |  |  |  |  |  |  |  |  |  |  |
| **Lomond (Lp)** | ***Na*** | 2.000 | 3.000 | 9.000 | 21.000 | 3.000 | 5.000 | 4.000 | 3.000 | 3.000 | 3.000 | 4.000 | 5.000 | 17.000 |  |
|  | ***Ar*** | 2.000 | 2.999 | 6.392 | 13.069 | 2.997 | 4.809 | 3.885 | 2.849 | 2.306 | 2.747 | 3.745 | 4.624 | 11.112 |  |
|  | ***Ho*** | 0.543 | 0.667 | 0.667 | 0.882 | 0.639 | 0.778 | 0.722 | 0.528 | 0.472 | 0.306 | 0.556 | 0.750 | 0.889 |  |
|  | ***He*** | 0.448 | 0.645 | 0.722 | 0.937 | 0.601 | 0.712 | 0.603 | 0.542 | 0.460 | 0.274 | 0.536 | 0.742 | 0.908 |  |
|  | ***p-value*** | 0.263 | 0.086 | 0.180 | 0.545 | 0.834 | 0.667 | 0.150 | 0.630 | 0.503 | 1.000 | 0.918 | 0.012 | 0.708 |  |
|  | ***Fis*** | -0.217 | -0.034 | 0.078 | 0.059 | -0.065 | -0.094 | -0.201 | 0.026 | -0.028 | -0.116 | -0.038 | -0.011 | 0.022 | -0.042 |
|  | ***Null alleles*** |  |  |  |  |  |  |  |  |  |  |  |  |  |  |
| **Bann (Lf Res)** | ***Na*** | 3.000 | 2.000 | 4.000 | 15.000 | 3.000 | 4.000 | 3.000 | 3.000 | 4.000 | 1.000 | 3.000 | 4.000 | 9.000 |  |
|  | ***Ar*** | 2.394 | 2.000 | 3.603 | 10.835 | 2.691 | 3.977 | 2.712 | 2.988 | 3.458 | 1.000 | 2.456 | 3.885 | 7.724 |  |
|  | ***Ho*** | 0.200 | 0.520 | 0.600 | 0.880 | 0.560 | 0.750 | 0.500 | 0.520 | 0.667 | mono | 0.250 | 0.625 | 0.917 |  |
|  | ***He*** | 0.220 | 0.393 | 0.588 | 0.908 | 0.528 | 0.689 | 0.551 | 0.598 | 0.680 | mono | 0.345 | 0.629 | 0.871 |  |
|  | ***p-value*** | 0.110 | 0.145 | 0.955 | 0.252 | 1.000 | 0.576 | 0.841 | 0.483 | 0.694 | mono | 0.322 | 0.248 | 0.237 |  |
|  | ***Fis*** | 0.091 | -0.333 | -0.021 | 0.031 | -0.062 | -0.091 | 0.094 | 0.133 | 0.020 | N.A. | 0.279 | 0.006 | -0.054 | -0.021 |
|  | ***Null alleles*** |  |  |  |  |  |  |  |  |  |  |  |  |  |  |
| **Scheldt (Lf)** | ***Na*** | 3.000 | 4.000 | 8.000 | 19.000 | 3.000 | 4.000 | 4.000 | 3.000 | 5.000 | 2.000 | 3.000 | 5.000 | 23.000 |  |
|  | ***Ar*** | 2.531 | 3.308 | 6.248 | 11.520 | 2.533 | 3.931 | 3.989 | 2.959 | 3.380 | 1.984 | 2.314 | 4.668 | 12.676 |  |
|  | ***Ho*** | 0.429 | 0.543 | 0.771 | 0.824 | 0.429 | 0.629 | 0.714 | 0.543 | 0.571 | 0.229 | 0.600 | 0.829 | 0.886 |  |
|  | ***He*** | 0.386 | 0.618 | 0.708 | 0.910 | 0.442 | 0.675 | 0.732 | 0.510 | 0.574 | 0.248 | 0.519 | 0.688 | 0.931 |  |
|  | ***p-value*** | 1.000 | 0.747 | 0.939 | 0.386 | 1.000 | 0.570 | 0.171 | 0.793 | 1.000 | 0.527 | 0.403 | 0.298 | 0.772 |  |
|  | ***Fis*** | -0.114 | 0.123 | -0.091 | 0.097 | 0.030 | 0.070 | 0.024 | -0.065 | 0.005 | 0.081 | -0.159 | -0.208 | 0.049 | -0.010 |
|  | ***Null alleles*** |  |  |  |  |  |  |  |  |  |  |  |  |  |  |
| **Trent (Lf)** | ***Na***  Cont. | 2.000 | 4.000 | 10.000 | 24.000 | 3.000 | 5.000 | 5.000 | 4.000 | 5.000 | 4.000 | 3.000 | 5.000 | 21.000 |  |
|  | ***Ar*** | 2.000 | 3.547 | 5.886 | 13.261 | 2.811 | 4.296 | 4.545 | 3.322 | 3.377 | 3.141 | 2.922 | 4.022 | 12.863 |  |
|  | ***Ho*** | 0.455 | 0.636 | 0.606 | 0.909 | 0.606 | 0.667 | 0.818 | 0.758 | 0.576 | 0.394 | 0.636 | 0.667 | 0.879 |  |
|  | ***He*** | 0.461 | 0.628 | 0.630 | 0.933 | 0.472 | 0.689 | 0.756 | 0.625 | 0.576 | 0.418 | 0.558 | 0.582 | 0.937 |  |
|  | ***p-value*** | 1.000 | 0.179 | 0.187 | 0.727 | 0.220 | 0.202 | 0.094 | 0.188 | 0.212 | 0.664 | 0.625 | 0.205 | 0.473 |  |
|  | ***Fis*** | 0.014 | -0.013 | 0.038 | 0.026 | -0.289 | 0.032 | -0.084 | -0.216 | 0.001 | 0.059 | -0.144 | -0.148 | 0.063 | -0.042 |
|  | ***Null alleles*** |  |  |  |  |  |  |  |  |  |  |  |  |  |  |

| **1** |  | 0 | 0.00909 | 0 | 0.7 | 0 | 0.02727 | 0 | 0.06364 | 0 | 0.03636 | 0.06364 | 0 | 0 | 0 | 0.43636 | 0 |  |
| --- | --- | --- | --- | --- | --- | --- | --- | --- | --- | --- | --- | --- | --- | --- | --- | --- | --- | --- |
| **2** | **0.10482** |  | 0 | 0 | 0 | 0 | 0 | 0 | 0 | 0 | 0 | 0 | 0 | 0 | 0 | 0 | 0 |  |
| **3** | **0.01467** | **0.12577** |  | 0 | 0.67273 | 0 | 0.02727 | 0 | 0.04545 | 0 | 0 | 0.06364 | 0 | 0 | 0 | 0.67273 | 0.37273 |  |
| **4** | **0.05688** | **0.14894** | **0.04958** |  | 0 | 0 | 0 | 0 | 0 | 0 | 0 | 0 | 0 | 0 | 0 | 0 | 0 |  |
| **5** | 0.00091 | **0.12279** | -0.00055 | **0.0752** |  | 0 | 0.91818 | 0 | 0.79091 | 0 | 0.92727 | 0.01818 | 0 | 0.27273 | 0 | 0.95455 | 0.98182 |  |
| **6** | **0.04408** | **0.16403** | **0.06178** | **0.14181** | **0.04307** |  | 0 | 0 | 0 | 0 | 0 | 0 | 0 | 0 | 0 | 0 | 0 |  |
| **7** | **0.00721** | **0.09472** | 0.00777 | **0.07476** | -0.00524 | **0.04667** |  | 0 | 0.82727 | 0 | 0.68182 | 0.03636 | 0 | 0 | 0 | 0.14545 | 0.56364 |  |
| **8** | **0.09712** | **0.191** | **0.08653** | **0.10329** | **0.09039** | **0.14962** | **0.08821** |  | 0 | 0 | 0 | 0 | 0 | 0 | 0 | 0 | 0 |  |
| **9** | 0.00077 | **0.09351** | 0.00525 | **0.0677** | -0.0049 | **0.04353** | -0.00379 | **0.0821** |  | 0 | 0.43636 | 0.02727 | 0 | 0 | 0 | 0.34545 | 0.77273 |  |
| **10** | **0.08193** | **0.17794** | **0.07584** | **0.11258** | **0.08824** | **0.13778** | **0.08315** | **0.16067** | **0.07846** |  | 0 | 0 | 0 | 0 | 0 | 0 | 0 |  |
| **11** | 0.00296 | **0.10022** | **0.01304** | **0.06757** | -0.00251 | **0.03921** | -0.00298 | **0.08703** | -0.00062 | **0.077** |  | 0 | 0 | 0 | 0 | 0.27273 | 0.2 |  |
| **12** | **0.0153** | **0.12324** | 0.00815 | **0.04645** | 0.01248 | **0.07604** | **0.01961** | **0.10592** | **0.01546** | **0.07703** | **0.02537** |  | 0 | 0.08182 | 0 | 0.30909 | 0.30909 |  |
| **13** | **0.08666** | **0.17404** | **0.09354** | **0.13216** | **0.0931** | **0.12483** | **0.08551** | **0.18227** | **0.07546** | **0.12966** | **0.08645** | **0.05919** |  | 0 | 0 | 0 | 0 |  |
| **14** | **0.01666** | **0.09681** | **0.02644** | **0.08605** | **0.011** | **0.06045** | **0.01438** | **0.11921** | **0.01153** | **0.07783** | **0.01928** | **0.01688** | **0.05341** |  | 0 | 0 | 0 |  |
| **15** | **0.05391** | **0.18554** | **0.04513** | **0.10832** | **0.04628** | **0.09256** | **0.04276** | **0.15284** | **0.05175** | **0.10987** | **0.05899** | **0.04383** | **0.11945** | **0.05999** |  | 0.00909 | 0 |  |
| **16** | -0.00082 | **0.0969** | -0.00072 | **0.05025** | -0.00333 | **0.05711** | -0.0003 | **0.07955** | -0.00265 | **0.08349** | 0.00041 | **0.00902** | **0.09156** | **0.019** | **0.04756** |  | 0.50909 |  |
| **17** | **0.01178** | **0.09953** | 0.00066 | **0.06703** | -0.0024 | **0.04391** | -0.00148 | **0.08412** | -0.00333 | **0.07572** | 0.00338 | **0.01406** | **0.0824** | **0.01783** | **0.05339** | -0.002 |  |  |
|  | **1** | **2** | **3** | **4** | **5** | **6** | **7** | **8** | **9** | **10** | **11** | **12** | **13** | **14** | **15** | **16** | **17** |  |

**Table S5.** Pairwise *F_ST_* values for 13 microsatellite loci. Grey represents *L. planeri*, blue represents *L. fluviatilis* and resident *L. fluviatilis* populations are shown in pink for ease of identification. Numbers in bold indicate a significance level of *P* < 0.05. 1: Wear (Lf); 2: Wear (Lp); 3: Dee (Lf); 4: Dee (Lp); 5: Derwent (Lf); 6: Derwent (Lp); 7: Nidd (Lf); 8: Nidd (Lp); 9: Ure (Lf); 10: Ure (Lp); 11: Swale (Lf); 12: Lomond (Lf anadromous); 13: Lomond (Lf freshwater-resident); 14: Lomond (Lp); 15: Bann (Lf freshwater – resident); 16: Scheldt (Lf); 17: Trent (Lf). Upper half of matrix shows the significance (*P*) values.

**Table S6**

| Migrate-N analysis (Bayesian) showing posterior distributions for 6 paired sites (95% confidence intervals in brackets). | | |
| --- | --- | --- |
|  | **M2-M1** | **M1-M2** |
| Dee | 12.49 (0.00 – 33.80) | 9.12 (0.00 – 31.20) |
| Wear | 9.40 (0.00 – 31.20) | 5.20 (0.00 – 26.00) |
| Ure | 9.43 (0.00 – 31.20) | 6.38 (0.00 27.73) |
| Derwent | 4.18 (0.00 – 27.07) | 3.73 (0.00 – 26.13) |
| Nidd | 16.28 (0.00 – 37.27) | 8.80 (0.00 – 30.33) |
| Lomond | 11.47 (0.00 – 32.93) | 10.43 (0.00 – 32.07) |
| *Note M1= *L. planeri* and M2=anadromous *L. fluviatilis.* For Loch Lomond the anadromous rather than the resident *L. fluviatilis* was included. | | |

**Table S7** Pairwise estimation of current gene flow, M, between (a) populations of anadromous *L. fluviatilis* (Lf) and *L. planeri* (Lp) from within the same river, and (b) populations within Loch Lomond by BAYESASS. Between species gene flow is highlighted in bold. Where M is the fraction of individuals in the ‘To’ population that are migrants originating in the ‘From’ population.

(a)

| ***From*** | ***To*** | **M +/- SD** | **95% CI** | |  |
| --- | --- | --- | --- | --- | --- |
| Dee Lf | Dee Lf | 0.6744**+/**-0.0075 | 0.660 - | 0.689 |  |
| **Dee Lf** | **Dee Lp** | **0.0071+/-0.0069** | -0.006 - | 0.021 |  |
| **Dee Lp** | **Dee Lf** | **0.0067+/-0.0065** | -0.006 - | 0.019 |  |
| Dee Lp | Dee Lp | 0.6749+/-0.0079 | 0.659 - | 0.690 |  |
| Wear Lf | Wear Lf | 0.6738+/-0.0069 | 0.660 - | 0.687 |  |
| **Wear Lf** | **Wear Lp** | **0.0070+/-0.0067** | -0.006 - | 0.020 |  |
| **Wear Lp** | **Wear Lf** | **0.0079+/-0.0077** | 0.064 - | 0.094 |  |
| Wear Lp | Wear Lp | 0.8355+/-0.1003 | 0.639 - | 1.032 |  |
| Derwent Lf | Derwent Lf | 0.6762+/-0.0091 | 0.658 - | 0.694 |  |
| **Derwent Lf** | **Derwent Lp** | **0.0075+/-0.0074** | -0.007 - | 0.022 |  |
| **Derwent Lp** | **Derwent Lf** | **0.0295+/-0.0171** | -0.004 - | 0.063 |  |
| Derwent Lp | Derwent Lp | 0.8951+/-0.0310 | 0.834 - | 0.956 |  |
| Nidd Lf | Nidd Lf | 0.6749+/-0.0080 | 0.659 - | 0.691 |  |
| **Nidd Lf** | **Nidd Lp** | **0.0071+/-0.0069** | -0.006 - | 0.021 |  |
| **Nidd Lp** | **Nidd Lf** | **0.0071+/-0.0069** | -0.006 - | 0.021 |  |
| Nidd Lp | Nidd Lp | 0.6749+/-0.0079 | 0.659 - | 0.690 |  |
| Ure Lf | Ure Lf | 0.6752+/-0.0082 | 0.659 - | 0.691 |  |
| **Ure Lf** | **Ure Lp** | **0.0077+/-0.0075** | -0.007 - | 0.022 |  |
| **Ure Lp** | **Ure Lf** | **0.0079+/-0.0078** | -0.007 - | 0.023 |  |
| Ure Lp | Ure Lp | 0.9000+/-0.253 | 0.404 | 1.396 |  |
|  |  |  |  |  | |

(b)

| ***From*** | ***To*** | **M +/- SD** | **95% CI** |
| --- | --- | --- | --- |

| *L. fluviatilis* | *L. fluviatilis* | 0.697 +/- 0.024 | 0.650 - 0.773 |
| --- | --- | --- | --- |
| ***L. fluviatilis*** | **Resident (Lf)** | **0.010 +/- 0.010** | **0 - 0.029** |
| ***L. fluviatilis*** | ***L. planeri*** | **0.011 +/- 0.011** | **0 - 0.032** |
| **Resident (Lf)** | ***L. fluviatilis*** | **0.117 +/- 0.032** | **0.055 - 0.179** |
| Resident (Lf) | Resident (Lf) | 0.969 +/- 0.017 | 0.937 - 1 |
| **Resident (Lf)** | ***L. planeri*** | **0.217 +/- 0.041** | **0.137 - 0.297** |
| ***L. planeri*** | ***L. fluviatilis*** | **0.186 +/- 0.037** | **0.113- 0.259** |
| ***L. planeri*** | **Resident (Lf)** | **0.021 +/- 0.014** | **0 - 0.048** |
| *L. planeri* | *L. planeri* | 0.772 +/- 0.040 | 0.693 - 0.851 |
|  |  |  |  |


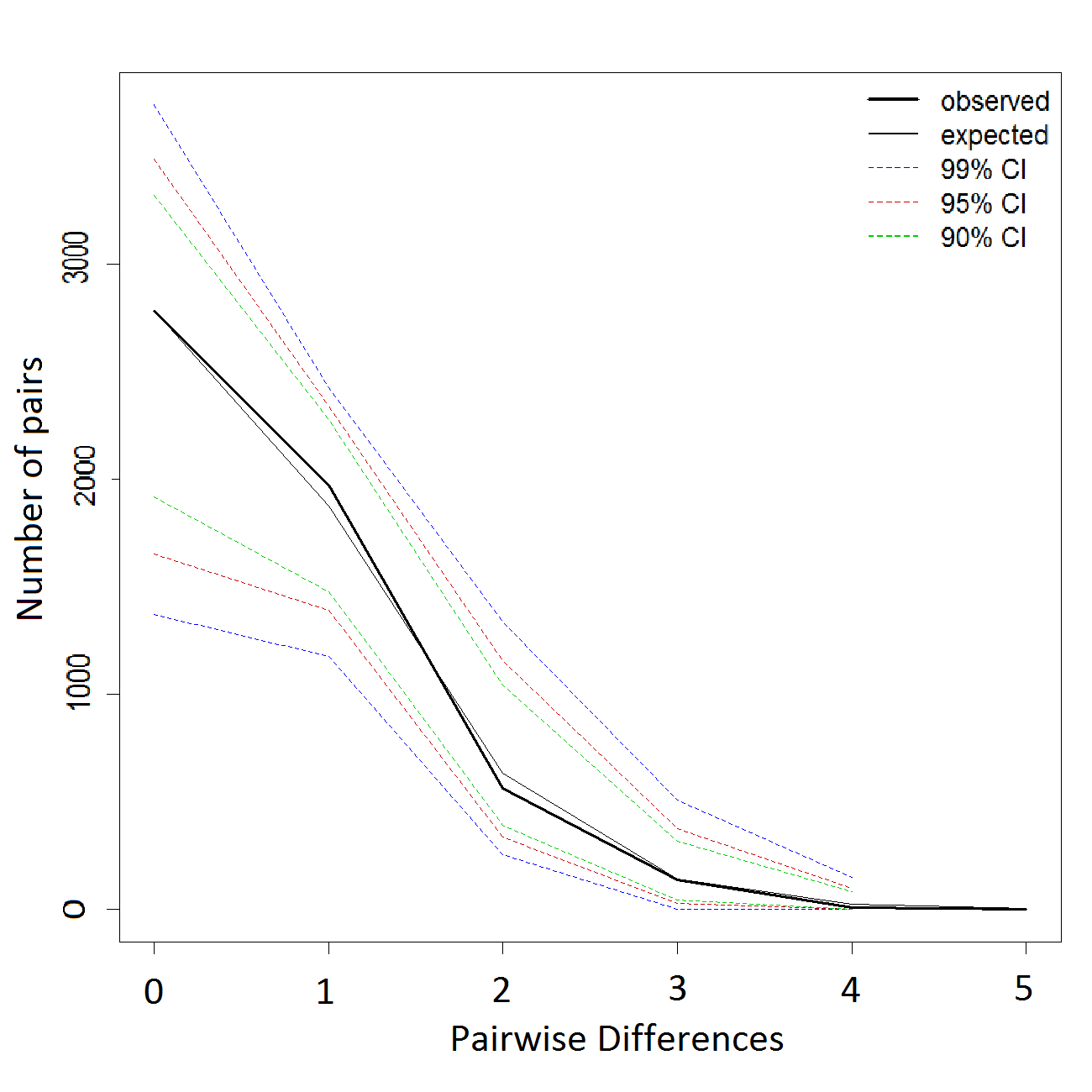


**Figure S1**: Mismatch Distribution (demographic expansion) with *Tau* 0.673, showing an expansion pattern for the six populations of *Lampetra fluviatilis* and *L. planeri* presented in Table 1.

|  |  |  |  |
| --- | --- | --- | --- |

**
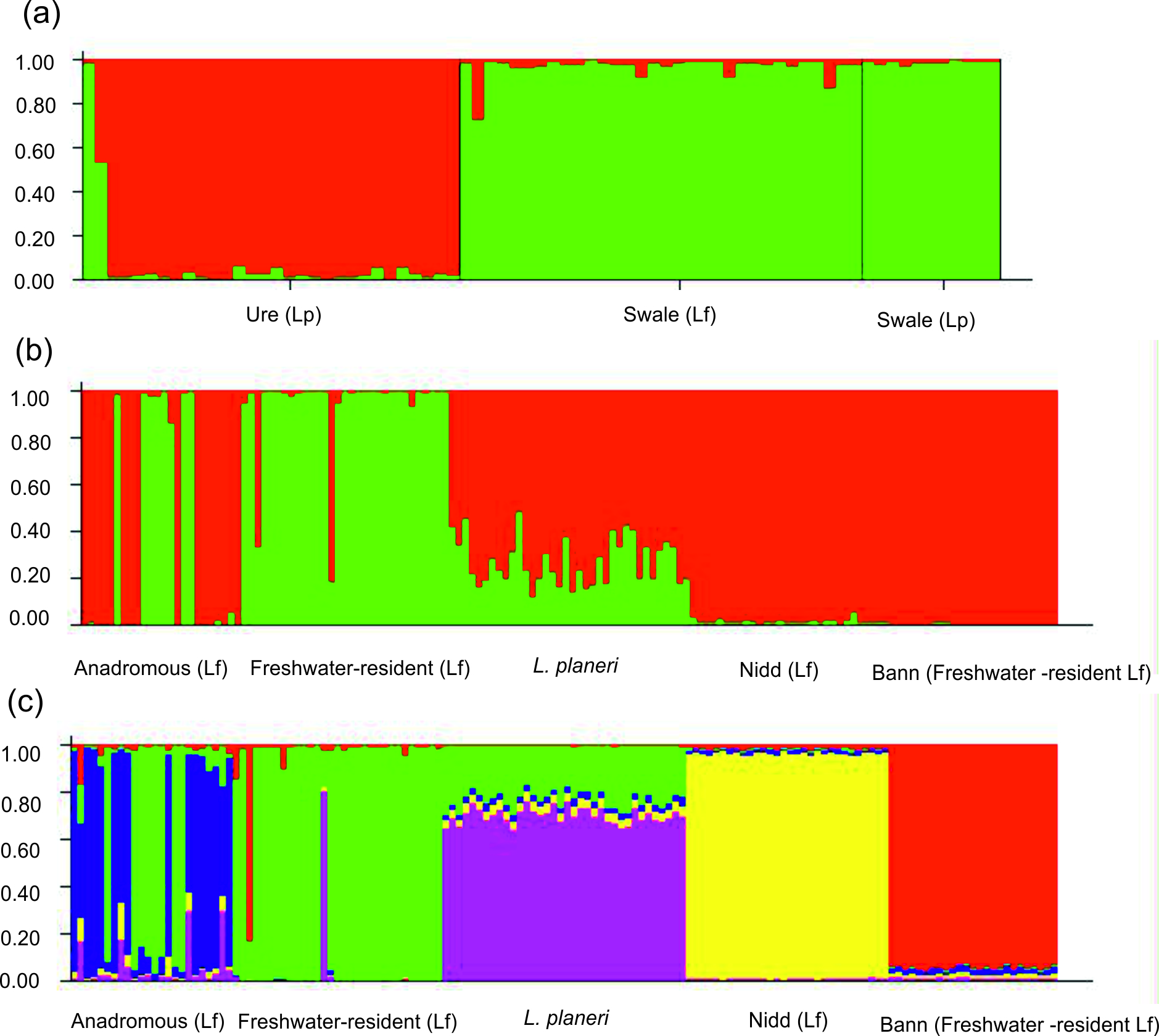
**

**Figure S2.** STRUCTURE bar plot generated from microsatellite data showing (a) *ΔK*= 2 (*LnP(D)=* -2294.8) where Swale Lp is compared to another *L. planeri* population and *L. fluviatilis* form the same river (b) *ΔK*= 2 when prior location information is used to analyse the Loch Lomond system which shows the freshwater-resident Lomond population to be differentiated and (c) *ΔK*= 5 (*LnP(D)=* -4681) when a location prior is used.


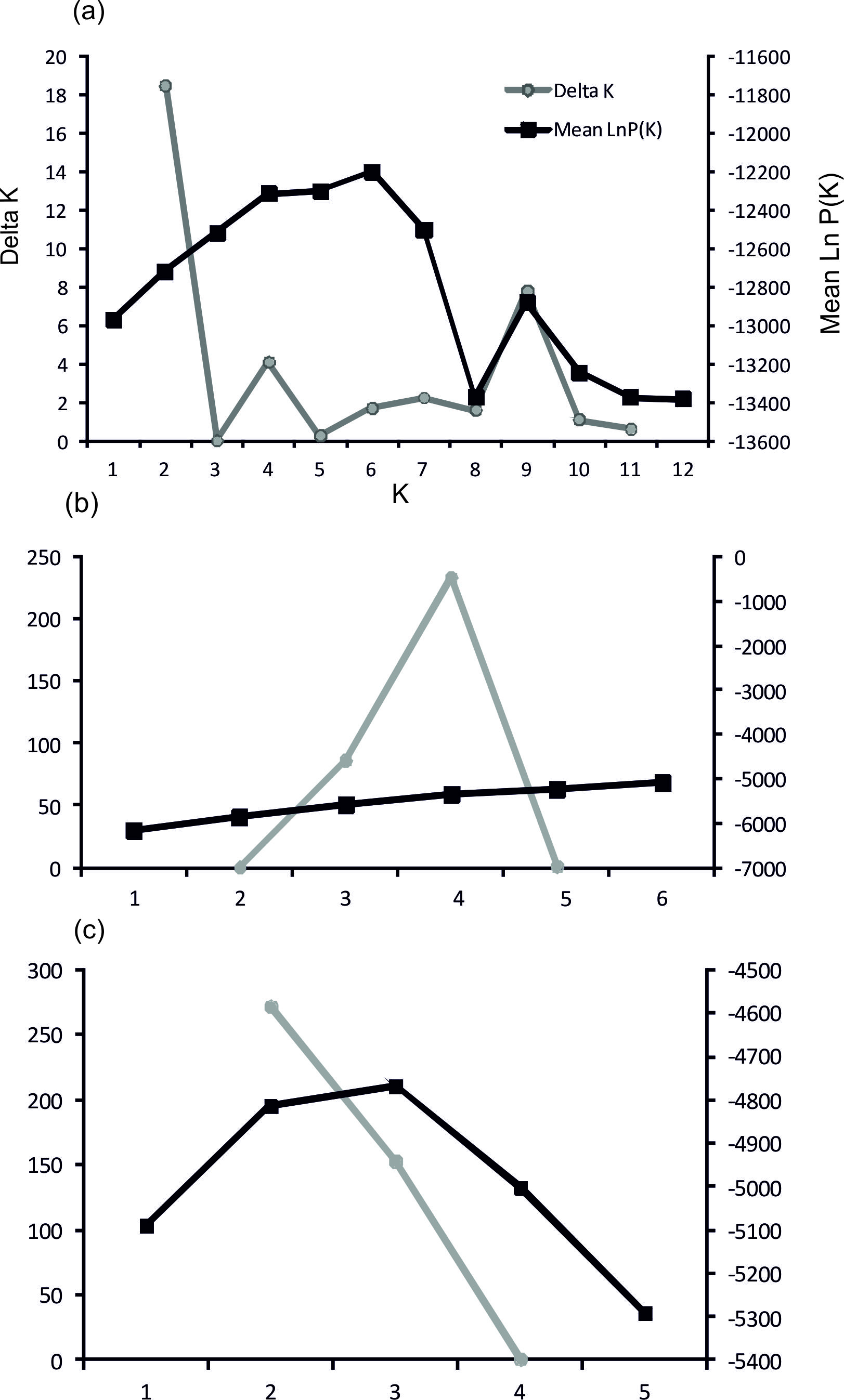


**Figure S3** Posterior probability of the data (Ln [P(D|K)]) and values of ΔK (Evanno *et al.* 2005) as a function of K (number of clusters), associated with the results shown in Figure 6 in the main text. (a) Comparison between *Lampetra fluviatilis* and *Lampetra planeri* populations (K=6), (b) *L. planeri* populations (K=6), (c) Loch Lomond populations compared to a population of *L. fluviatilis*  from the Humber catchment and freshwater-resident *L. fluviatilis* populations from the R. Bann in N. Ireland (K=3).

**a)**

**
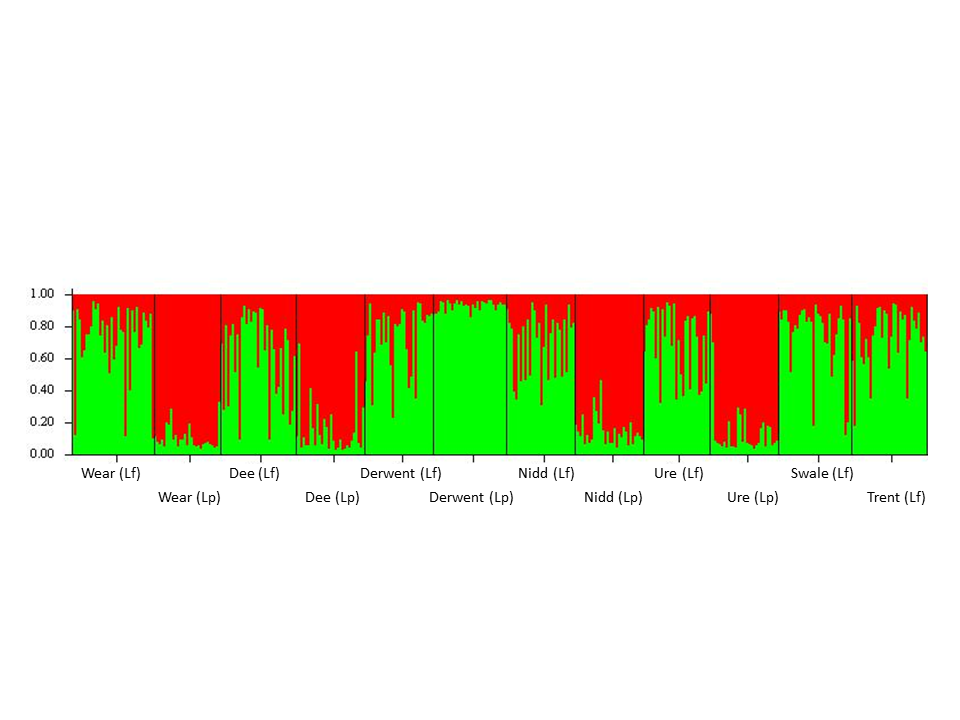
**

**b)**

**
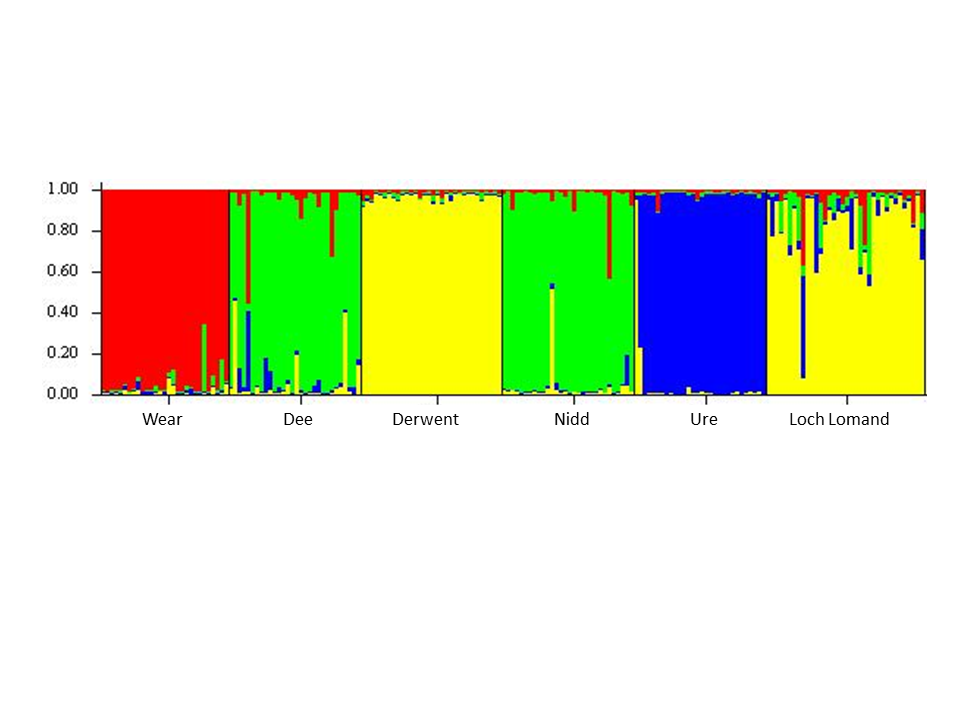
**

**c)**

**
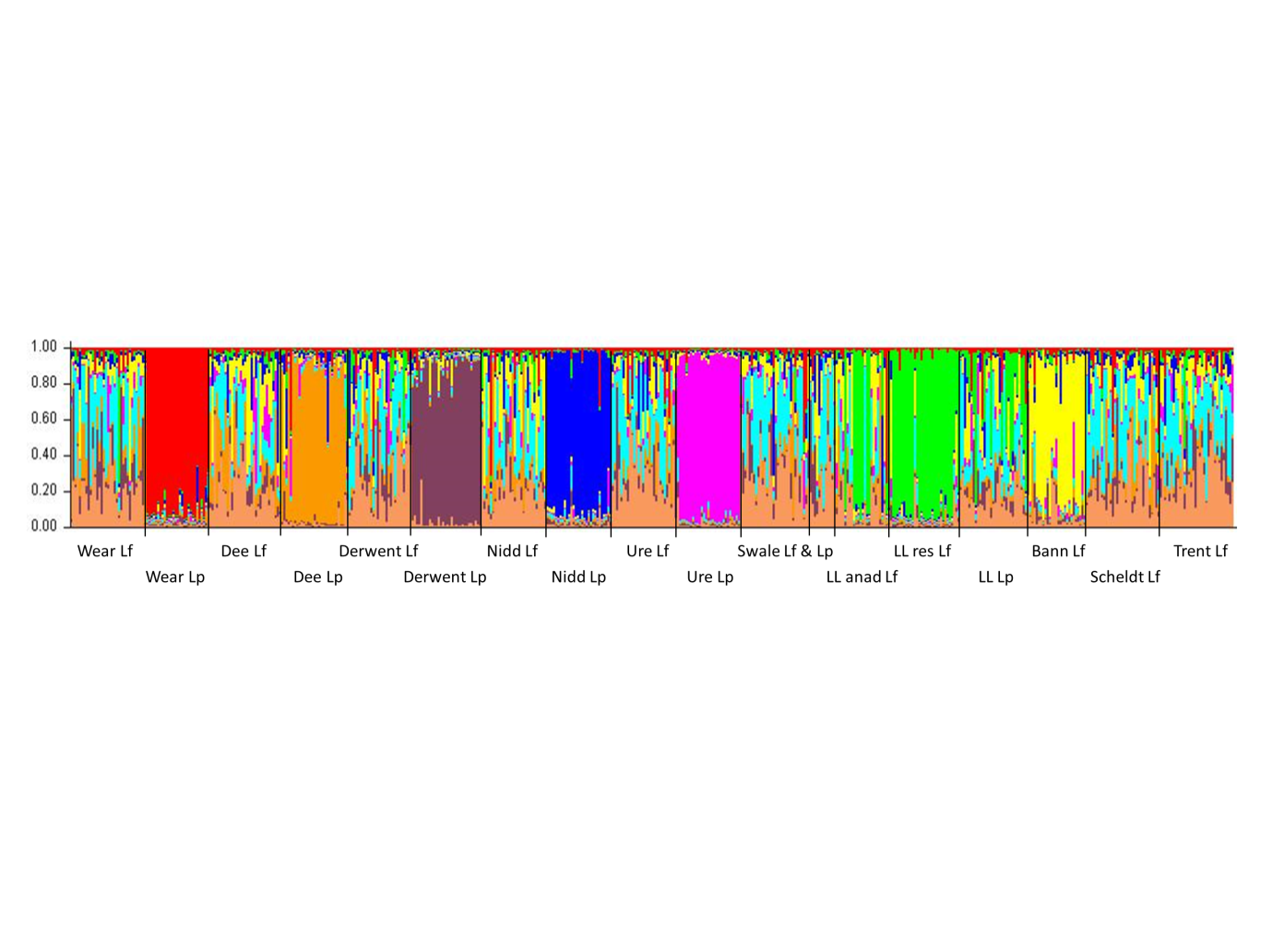
**

**d)**

**
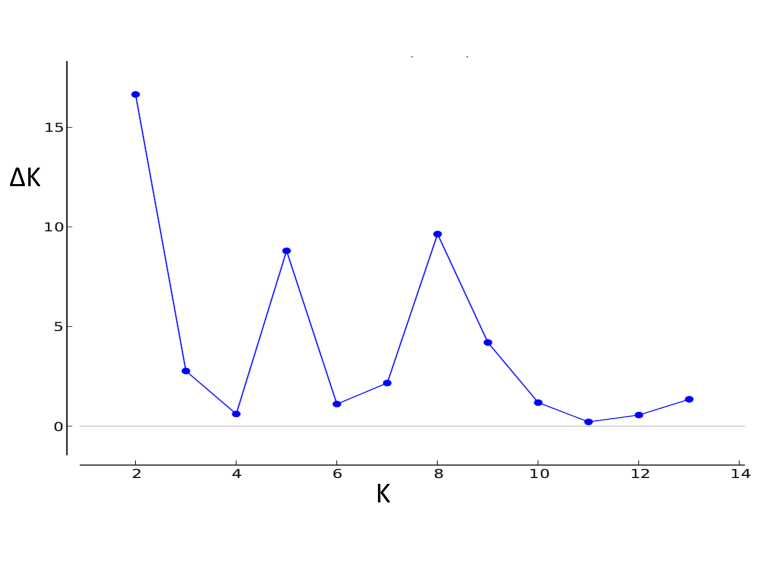
**

**Figure S4**: a) Structure for 12 populations where ∆K=2. b) Structure for *L. planeri* only where ∆K = 4. c) Structure for all samples included when K=10 (Lf = *L. fluviatilis*, Lp = *L. planeri*, LL= Loch Lomond; anad = anadromous; res = resident). d) ∆K plot for the structure plot in part c.


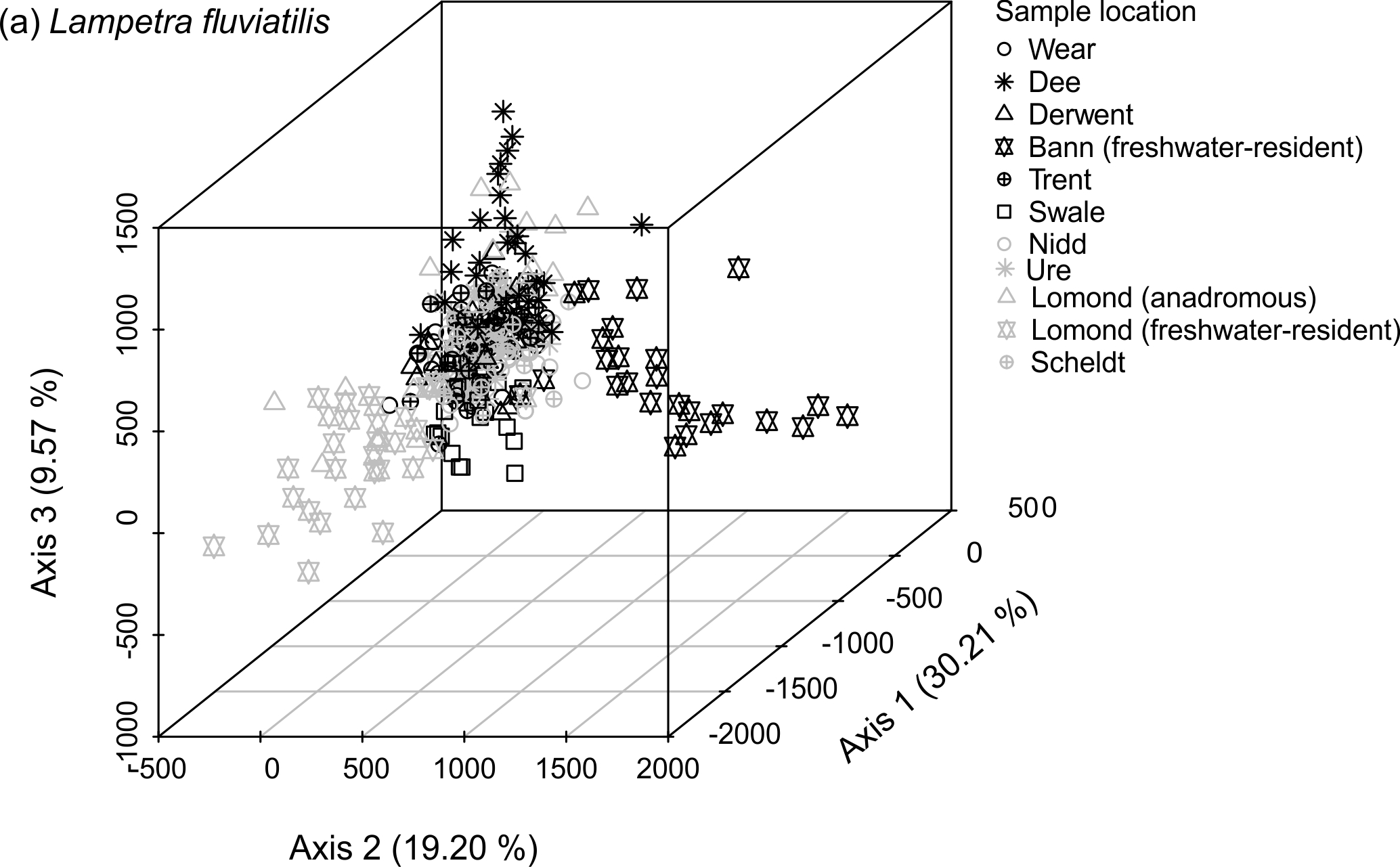


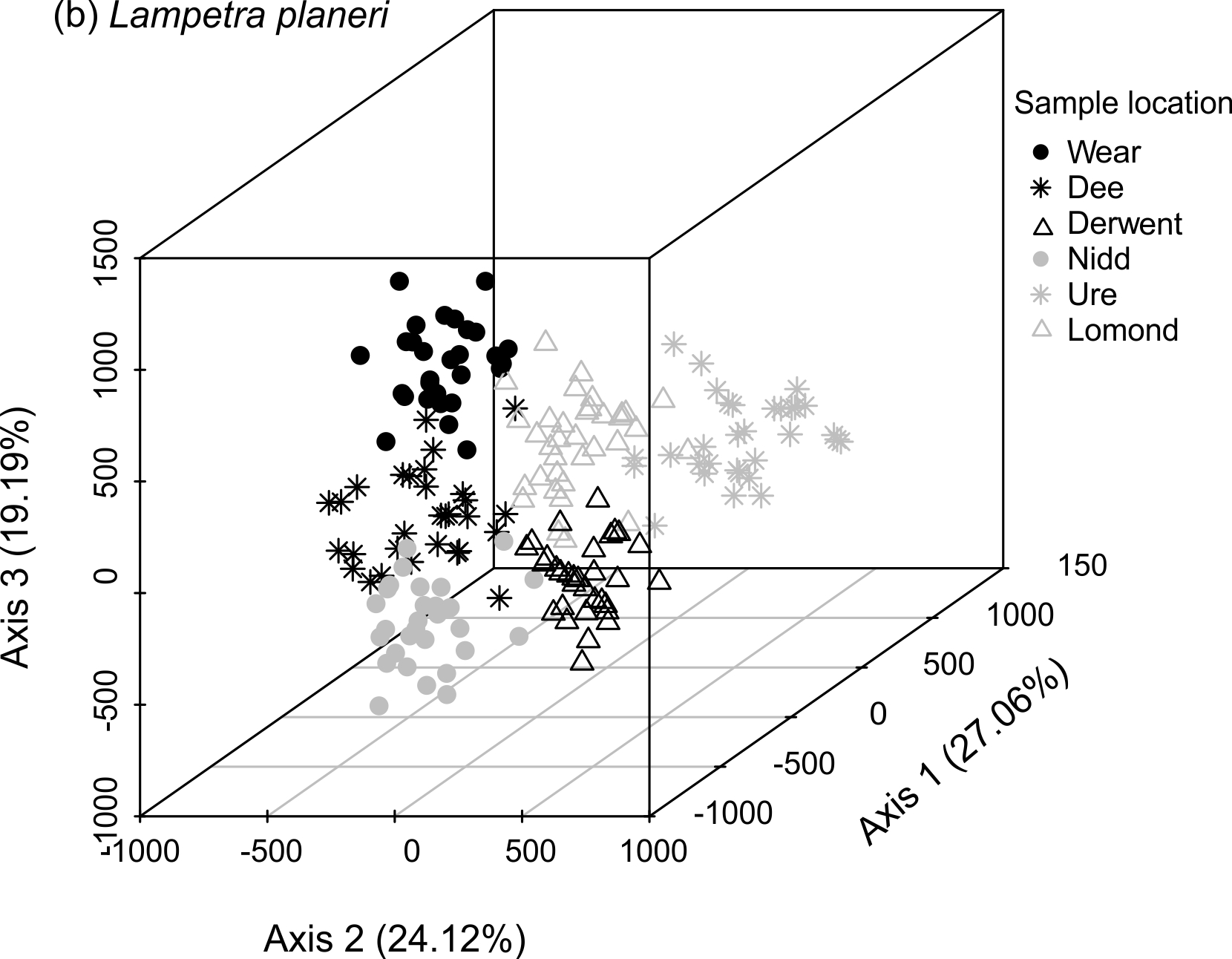


**Figure S5.** FCA analysis for (a) *Lampetra fluviatilis* and (b) *Lampetra planeri* population
